# Adipose tissue NAPE-PLD controls fat mass development by altering the browning process and gut microbiota

**DOI:** 10.1038/ncomms7495

**Published:** 2015-03-11

**Authors:** Lucie Geurts, Amandine Everard, Matthias Van Hul, Ahmed Essaghir, Thibaut Duparc, Sébastien Matamoros, Hubert Plovier, Julien Castel, Raphael G. P. Denis, Marie Bergiers, Céline Druart, Mireille Alhouayek, Nathalie M. Delzenne, Giulio G. Muccioli, Jean-Baptiste Demoulin, Serge Luquet, Patrice D. Cani

**Affiliations:** 1 Metabolism and Nutrition Research Group, WELBIO-Walloon Excellence in Life Sciences and BIOtechnology, Louvain Drug Research Institute, Université catholique de Louvain, Avenue E. Mounier, 73 B1.73.11, 1200 Brussels, Belgium; 2 de Duve Institute, Université catholique de Louvain, Avenue Hippocrate, 74 B1.74.05, 1200 Brussels, Belgium; 3 Université Paris Diderot, Sorbonne Paris Cité, BFA, UMR8251, CNRS, F-75205 Paris, France; 4 Bioanalysis and Pharmacology of Bioactive Lipids Research Group, Louvain Drug Research Institute, Université catholique de Louvain, Avenue E. Mounier, 72 B1.72.11, 1200 Brussels, Belgium

## Abstract

Obesity is a pandemic disease associated with many metabolic alterations and involves several organs and systems. The endocannabinoid system (ECS) appears to be a key regulator of energy homeostasis and metabolism. Here we show that specific deletion of the ECS synthesizing enzyme, NAPE-PLD, in adipocytes induces obesity, glucose intolerance, adipose tissue inflammation and altered lipid metabolism. We report that *Napepld*-deleted mice present an altered browning programme and are less responsive to cold-induced browning, highlighting the essential role of NAPE-PLD in regulating energy homeostasis and metabolism in the physiological state. Our results indicate that these alterations are mediated by a shift in gut microbiota composition that can partially transfer the phenotype to germ-free mice. Together, our findings uncover a role of adipose tissue NAPE-PLD on whole-body metabolism and provide support for targeting NAPE-PLD-derived bioactive lipids to treat obesity and related metabolic disorders.

## Abstract (web summary)

Endocannabinoids are bioactive lipid molecules produced in the body. Here, Geurts *et al*. create mice lacking the endocannabinoid-producing enzyme NAPE-PLD in adipocytes and report defects in adipose-induced browning, which are mediated by alterations in the gut microbiome.

## Graphical abstract

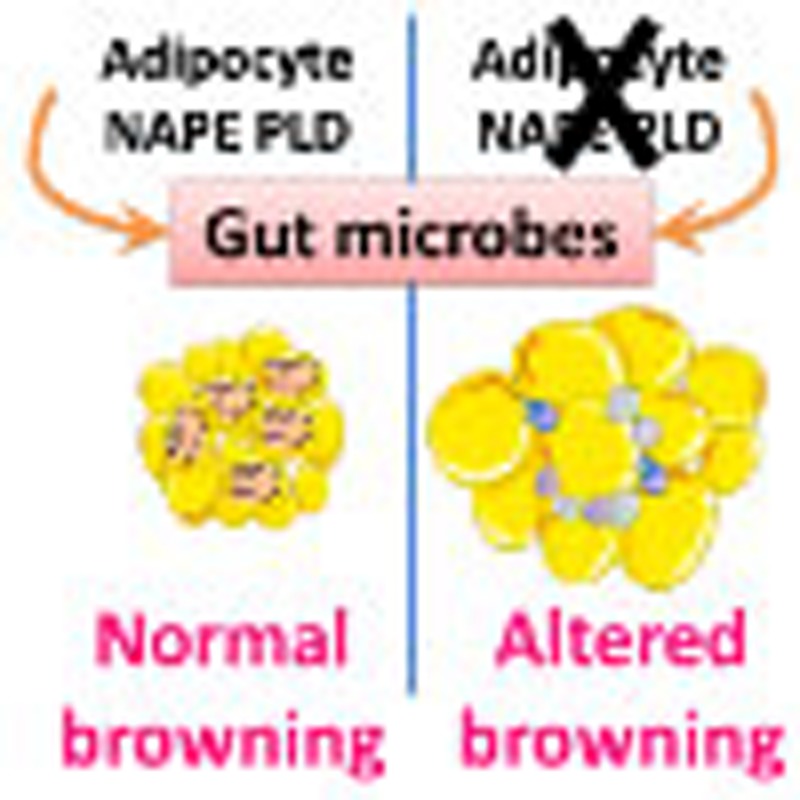

Obesity has reached pandemic levels. In addition to being associated with a massive expansion of adipose tissue, obesity is also associated with a cluster of metabolic alterations, such as type 2 diabetes and cardiovascular and hepatic diseases. Thus, it is of the utmost importance to unravel the underlying mechanisms that lead to these metabolic alterations to discover new therapeutic strategies. Obesity can be considered a ‘multi-system disease’ because several organs and systems participate in this metabolic condition. Among those, the endocannabinoid system (ECS) appears to be a key regulator of energy homeostasis and metabolism.

The ECS is a complex system composed of several bioactive lipids interacting with both membrane-bound and nuclear receptors, leading to a broad range of physiological effects. ECS activity is mainly controlled by key synthesis and degradation enzymes^1^. Anandamide (*N*-arachidonoylethanolamine, AEA) is one of the best characterized endocannabinoids (eCBs) and is involved in regulation of appetite and energy homeostasis^2^,^3^. In addition to AEA, other related *N*-acylethanolamines (NAEs), such as *N*-palmitoylethanolamine (PEA), *N*-stearoylethanolamine (SEA) and *N*-oleoylethanolamine (OEA) share biosynthetic and degrading pathways with AEA. NAEs are typically synthesized by the enzyme *N*-acylphosphatidylethanolamine phospholipase D (NAPE-PLD), although alternative pathways exist for AEA biosynthesis^1^,^4^. The eCBs are synthesized from cell membrane phospholipids and are released to the extracellular compartment to target their receptors. eCBs act via an autocrine or a paracrine mechanism^5^. The principal eCB receptors are the G-coupled receptors CB_1_ and CB_2_, which are mainly targeted by AEA and 2-arachidonoylglycerol, the two major eCBs. Other NAEs, such as OEA or PEA, activate non-cannabinoid receptors, including PPARα, GPR55 or GPR119 (refs 1, 6, 7, 8). The levels of eCBs are closely regulated by a balance between synthesis and degradation. After release, eCBs and NAEs are rapidly degraded by a cluster of degrading enzymes such as fatty acid amide hydrolase (FAAH) or NAE hydrolyzing acid amidase (NAAA)^5^. Because they are primarily synthesized on demand, it is worthwhile to focus on eCB production. NAEs modulate food intake and inflammatory response^3^,^9^,^10^ and thus seem to act as important mediators of metabolic homeostasis and inflammation. Studies on total *Napepld* knockout (KO) mice revealed no overt phenotype, highlighting alternative synthesis pathways for certain long-chain NAEs such as AEA and suggesting a role for NAPE-PLD in regulating lipid signalling systems^4^,^11^. These studies focused on the central ECS, but the exact role of NAPE-PLD *per se* in metabolism in peripheral tissues has yet to be investigated.

Among the peripheral tissues involved in obesity, the adipose tissue plays a central role. Besides storing excessive energy, the adipose tissue is an active metabolic organ that secretes many mediators, such as hormones and cytokines (for example, adipokines)^12^,^13^. We and others have previously underlined a role for the ECS in adipogenesis and adipose tissue function^14^,^15^,^16^,^17^, thereby designating the ECS as an important actor in adipose tissue metabolism. We hypothesize that NAEs produced by adipocytes are key mediators regulating whole-body metabolism and energy homeostasis.

To evaluate the specific role of NAEs produced in adipose tissue, we generated a mouse model of adipocyte-specific deletion of the *Napepld* gene and investigated the physiological role of adipose tissue NAPE-PLD under basal (control diet (CT)) and pathological (diet-induced obesity (DIO)) conditions. We found in this study that *Napepld* deletion in adipose tissue leads to development of obesity, impairment of glucose and lipid homeostasis along with altered adipose tissue metabolism and changes in gut microbiota composition.

## Results

### *Napepld* deletion is specific of adipose tissue

**Figure 1:**
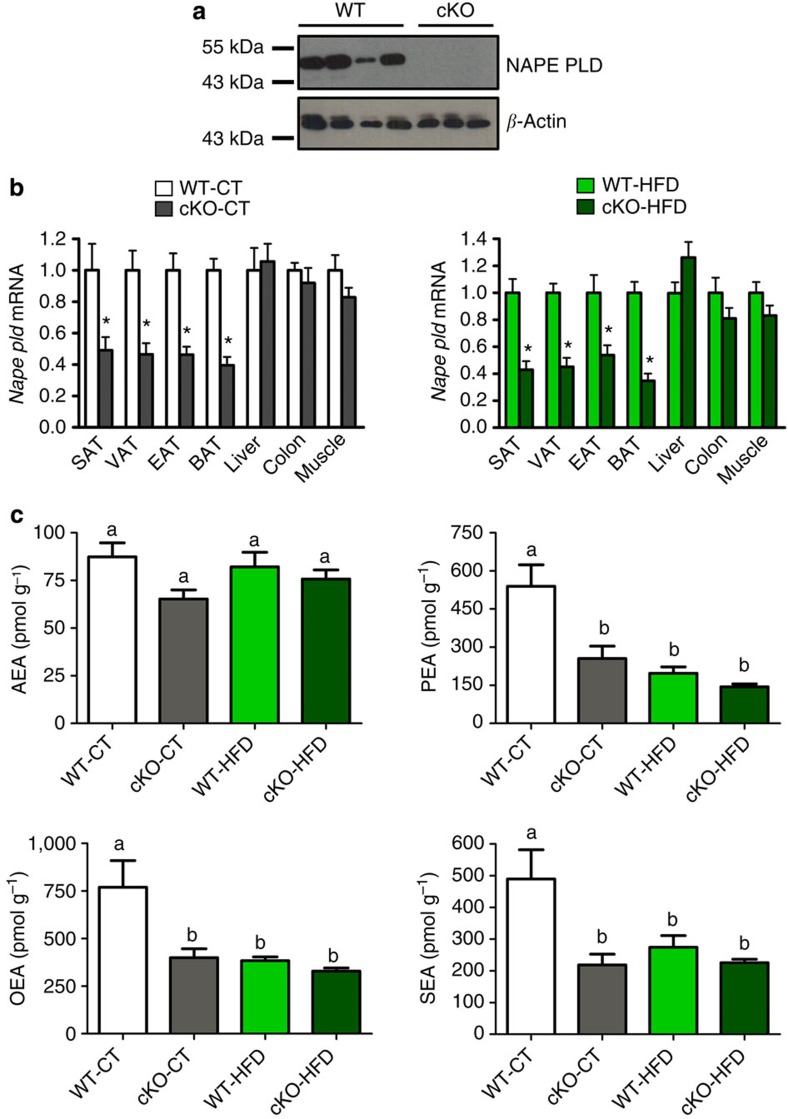
Specific deletion of *Napepld* in adipose tissue. (**a**) Representative adipose tissue immunoblot of NAPE-PLD and β-actin in WT mice and cKO mice. (**b**) mRNA relative expression of *Napepld* in different adipose tissue deposits (subcutaneous, visceral, epididymal and brown adipose tissue—SAT, VAT, EAT and BAT), as well as in liver, colon and tibialis muscle under CT diet and HFD in cKO mice and WT mice (*n*=20–27). These data (**a**,**b**) correspond to the results of three independent experiments. Data with ‘*’ indicate a significant difference (*P<*0.05) versus WT-CT or WT-HFD according to the unpaired two-tailed Student’s *t*-test. (**c**) SAT levels of AEA, PEA, OEA and SEA (pmol per g tissue) measured by HPLC-MS (*n*=6–10). Data are presented as the mean±s.e.m. Data with different superscript letters are significantly different (*P<*0.05) according to *post-hoc* one-way analysis of variance statistical analysis.

To assess the role of adipose tissue NAPE-PLD on metabolism, *Napepld*^*lox/lox*^ mice (construction in Supplementary Fig. 1) were crossed with *Fabp4-Cre* mice to generate mice with a conditional adipocyte-specific KO (cKO) of NAPE-PLD. *Fabp4-Cre*-*Napepld c*KO mice have a normal postnatal development, in contrast to other *Fabp4-Cre* mice strains that can develop postnatal lethality^18^. To confirm the invalidation of the *Napepld* gene in the adipose tissue of the cKO mice, we assessed the presence of the NAPE-PLD protein by Western blot analysis in the white adipose tissue (WAT) of wild-type (WT) and cKO mice (Fig. 1a) and found no detectable amounts of NAPE-PLD in the WAT of cKO mice. In contrast, we did not observe reduced NAPE-PLD levels in the brain, which demonstrates the specificity of our model (Supplementary Fig. 2). In addition, the analysis of messenger RNA (mRNA) expression from multiple tissues confirms that the deletion is specific for different depots of WAT (subcutaneous, visceral and epididymal) and brown adipose tissue (BAT; Fig. 1b), without affecting *Napepld* expression in the liver, colon or muscles, which indicates that recombination did not occur in other tissues^19^. During experiments, WT and cKO mice were fed either a CT (WT-CT and cKO-CT groups) or a high fat diet (HFD; WT-HFD and cKO-HFD groups). Deletion of the *Napepld* gene was verified in cKO groups under both diets (Fig. 1b). Because we observed a residual expression of *Napepld* in the adipose tissue, we performed a separation of the stromal vascular fraction (SVF) and adipocytes enriched fraction in the WAT. This indicated that decreased expression of *Napepld* occurs only in adipocytes fraction and not in the SVF (Supplementary Fig. 2). Some reports in the literature established a *Cre* activity mediated by the *Fabp4* promoter in other cell types such as macrophages^20^. To verify *Napepld* expression in macrophages, we isolated peritoneal macrophages from WT and cKO mice. We found that macrophages from both genotypes did not differ in *Napepld* expression (Supplementary Fig. 2). Finally, to ensure that the deletion of *Napepld* is indeed reducing NAE levels we measured the levels of NAEs produced by NAPE-PLD. Figure 1c illustrates ~60% reduction of PEA, OEA and SEA levels in the adipose tissue of cKO mice compared with WT mice. In contrast, we found no decrease in NAEs levels in the brain when comparing both genotypes (Supplementary Fig. 2). The lack of a significant impact of *Napepld* deletion on AEA confirms the existence of an alternative synthesis pathway for this NAE^4^,^11^,^21^. Importantly, we determined that HFD-treated WT mice exhibited similar levels of NAEs to cKO mice, suggesting that HFD treatment in itself has a NAE lowering effect that was only slightly intensified by the cKO genotype. Moreover, we found that *Napepld* deletion in adipose tissue leads to increased NAE precursor levels (that is, NAPEs) in adipose tissue, corroborating results of previous studies performed in *Napepld*^*−/−*^ mice^4^,^11^,^21^ (Supplementary Fig. 2).

### Adipose tissue *Napepld*-deleted mice develop an obese phenotype

**Figure 2:**
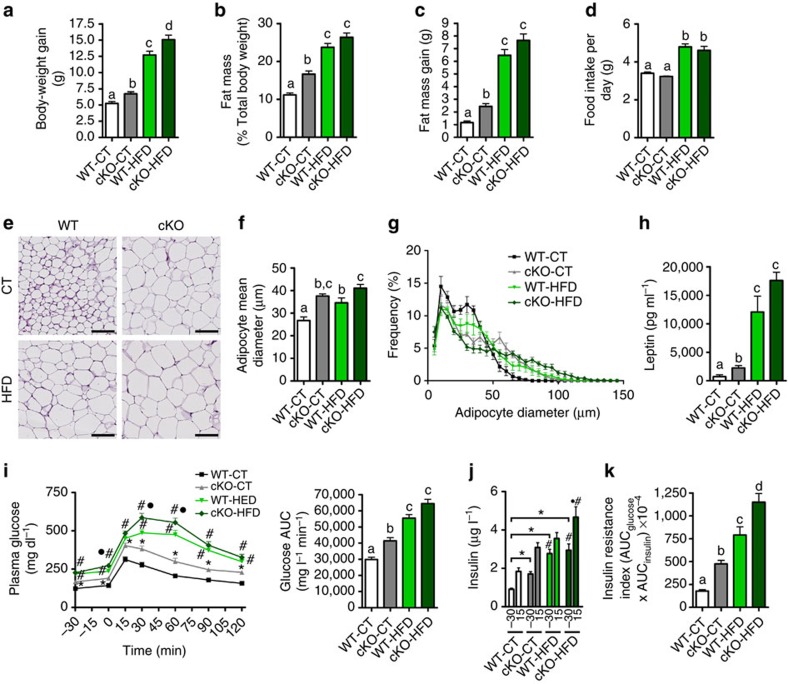
Adipose tissue *Napepld* deletion induces an obese-like phenotype. (**a**) Total body-weight gain (g) (*n*=20–27). (**b**) Total fat mass (% of total body weight) measured by TD-NMR (*n*=20–27). (**c**) Total fat mass gain (g) measured by TD-NMR (*n*=20–27). (**d**) Mean daily food intake per mouse (g) (*n*=20–27). (**e**) Representative haematoxylin and eosin-stained pictures of SAT deposits. Scale bar, 100 μm. (*n*=6–10). (**f**) Mean adipocyte diameter (μm) determined by histological analysis (*n*=6–10). (**g**) Adipocyte distribution and frequency with respect to the mean diameter measured by histological analysis (*n*=6–10). (**h**) Leptin plasma levels measured in the vena cava (μg ml^−1^; *n*=6–10). (**i**) Plasma glucose (mg dl^−1^) profile and the mean area under the curve (AUC) measured between 0 and 120 min after glucose loading (mg dl^−1^ min; *n*=20–27). (**j**) Plasma insulin levels at 30 min before and 15 min after glucose loading (*n*=20–27). (**k**) Insulin resistance index determined by multiplying the AUC of blood glucose by the AUC of insulin. These data (**a**–**d** and **i**–**k**) correspond to the results of three independent experiments. Data are presented as the mean±s.e.m. Data with different superscript letters are significantly different (*P<*0.05) according to *post-hoc* one-way analysis of variance (**a**–**h** and **k**). ‘*’ indicates a significant difference versus WT-CT (*P<*0.05) (**i**) and at both time points (−30 and 15) versus WT-CT (*P<*0.05) (**j**); ‘#’ indicates a significant difference (*P<*0.05) versus WT-CT and cKO-CT; and ‘·’ indicates a significant difference versus WT-HFD as determined by a two-way statistical analysis (**i,j**).

Surprisingly, under the CT diet, cKO mice gained more weight than WT mice, a phenomenon exacerbated under the HFD (Fig. 2a). Body composition measured by NMR indicated that the cKO mice accumulate more fat mass, which results in a higher percentage of total body fat and increased fat mass gain (Fig. 2b,c) and a lower percentage of lean mass when compared with their control counterparts after 8 weeks of CT diet (Supplementary Fig. 3). Food intake remains unchanged between the WT and cKO mice but is increased in the HFD-treated groups compared with the CT-treated groups (Fig. 2d). The increase in fat mass gain observed in cKO mice is associated with an increase in adipocyte size in both mice on the CT diet and HFD (Fig. 2e,f). This increase is reflected by a higher frequency of larger adipocytes in cKO mice and HFD mice compared with WT-CT mice (Fig. 2g). Moreover, the plasma levels of leptin, produced proportionally to fat mass, are markedly increased in cKO-CT mice compared with WT-CT mice and are increased even more in cKO-HFD mice compared with WT-HFD mice (Fig. 2h). We next assessed whether adipose *Napepld* deletion has an impact on whole-body glucose metabolism. We observed that cKO-CT mice are hyperglycemic in the fasted state and that these mice develop glucose intolerance, as evidenced by an oral glucose tolerance test (OGTT) (Fig. 2i). Importantly, this glucose intolerance is maintained throughout the duration of the OGTT. In addition, adipose *Napepld* deletion exacerbated HFD-induced glucose intolerance (Fig. 2i). The cKO-CT mice exhibit a twofold higher level of plasma insulin in the fasted state as well as after the oral glucose load, and this latter effect is also present during HFD feeding (Fig. 2j). These observations are confirmed by the increased insulin resistance index observed in cKO-CT during both CT and HFD diet feeding, the latter being worsened in cKO-HFD mice compared with WT-HFD mice (Fig. 2k).

### Adipose tissue *Napepld* deletion induces insulin resistance

**Figure 3:**
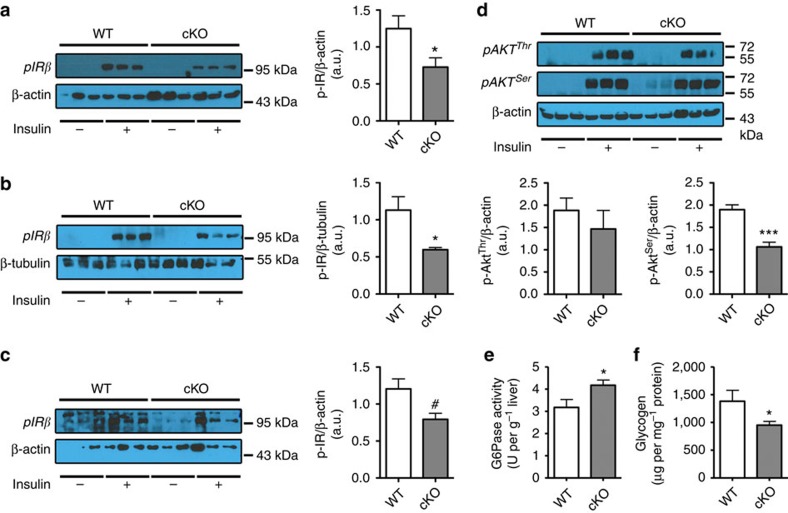
Adipose tissue *Napepld* deletion induces insulin resistance in the liver. (**a**) Representative liver immunoblots for p-IRβ and β-Actin in WT and cKO mice with or without insulin stimulation. Ratio of the insulin-stimulated p-IRβ in the liver on the loading control as measured by densitometry in cKO and WT mice (*n*=6). (**b**) Representative skeletal muscle immunoblots for p-IRβ and β-tubulin in WT and cKO mice with or without insulin stimulation. Ratio of the insulin-stimulated p-IRβ in the muscle on the loading control as measured by densitometry in cKO and WT mice (*n*=3–6). (**c**) Representative adipose tissue immunoblots, for p-IRβ and β-Actin in WT and cKO mice with or without insulin stimulation. Ratio of the insulin-stimulated p-IRβ in the adipose tissue on the loading control as measured by densitometry in cKO and WT mice (*n*=3). (**d**) Representative liver immunoblots for p-Akt^Thr308^, p-Akt^Ser473^ and β-Actin in WT and cKO mice with or without insulin stimulation. Ratio of the insulin-stimulated p-Akt^Thr308^ and p-Akt^Ser473^ in the liver on the loading control as measured by densitometry in cKO and WT mice (*n*=6). (**e**) Liver G6Pase activity (U per g liver) (*n*=6–10). (**f**) Liver glycogen content (μg per mg proteins; *n*=6–10). Data are presented as mean±s.e.m. Data with ‘*’ are significantly different (*P<*0.05), data with ‘***’ are significantly different (*P<*0.001), data with ‘#’ tend to be significantly different (*P=*0.06) according to the unpaired two-tailed Student *t*-test.

Insulin resistance in cKO mice is suggested by the increased fasted glycaemia, glucose intolerance, fasted and fed hyperinsulinemia and a higher insulin resistance index (Fig. 2i–k). To explore which organ may be responsible for insulin resistance, we analyzed insulin-induced phosphorylation of the insulin receptor (p-IRβ). We found that following insulin stimulation, phosphorylation of IRβ was strongly reduced in the liver and in the muscles of cKO mice, whereas the reduced phosphorylation of IRβ in adipose tissue was not statistically significant (Fig. 3a–c). To further analyze insulin resistance in the liver, we next analyzed insulin-induced phosphorylation of Akt (p-Akt), downstream mediator in the insulin signalling pathway. Phosphorylation of Akt on the serine site after insulin stimulation was reduced in the liver (Fig. 3d), confirming insulin resistance in this organ, whereas p-Akt levels were not affected in muscle or adipose tissue (data not shown). Finally, we found that glucose-6-phosphatase activity was increased and glycogen content decreased in the liver, confirming insulin resistance in this organ (Fig. 3e,f).

### *Napepld* deletion impacts adipose and whole-body lipid profiles

**Figure 4:**
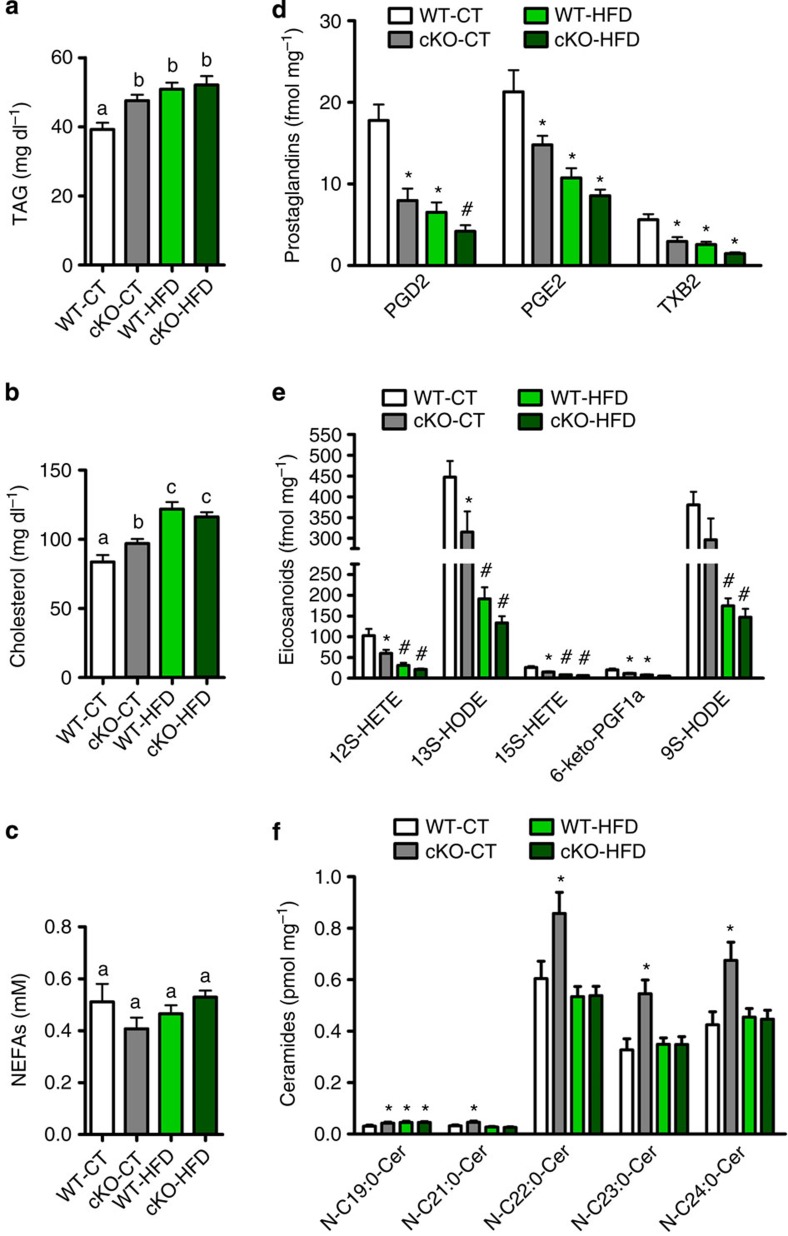
Adipose tissue *Napepld* deletion alters adipose and whole-body lipid metabolism. (**a**) Plasma triglyceride content (mg dl^−1^; *n*=20–27). (**b**) Plasma cholesterol content (mg dl^−1^; *n*=20–27). (**c**) Plasma non-esterified fatty acid (NEFA) levels (mM; *n*=20–27). These data (**a**–**c**) correspond to the results of three independent experiments. (**d**) SAT prostaglandin content measured by LC-MS/MS in fmol per mg tissue (*n*=10). (**e**) SAT eicosanoid content measured by LC-MS/MS in fmol per mg tissue (*n*=10). (**f**) SAT ceramide content measured by ESI-MS/MS in pmol per mg tissue (*n*=10). See also Supplementary Table 1. Data are presented as mean±s.e.m. Data with different superscript letters or symbols are significantly different (*P<*0.05) according to *post-hoc* one-way analysis of variance. ‘*’ indicates a significant difference (*P<*0.05) versus WT-CT, and ‘#’ indicates a significant difference (*P<*0.05) versus WT-CT and cKO-CT according to *post-hoc* one-way analysis of variance.

*Napepld* deletion leads to decreased synthesis of bioactive mediators (that is, NAE) which could be involved in the regulation of lipid synthesis and release by the adipose tissue. To examine this possibility, we performed an analysis of circulating lipids and adipose tissue lipidomics. We observed increased circulating triglycerides (TAG) and cholesterol levels (Fig. 4a,b) in cKO mice, whereas circulating non-esterified fatty acids levels were not affected by the deletion (Fig. 4c). In addition, NAEs share similar biosynthetic pathways with eicosanoids and their derivatives (namely prostaglandins (PG)), which are also important lipid mediators involved in metabolism and inflammation^22^. As we cannot exclude the possibility that adipose tissue *Napepld* deletion also affects these lipid mediators and that this interaction could be an additional mechanism involved in changes in energy homeostasis and glucose metabolism, we quantified the adipose levels of eicosanoids, PG, ceramides and phospholipids using a lipidomic approach. We observed that the deletion of *Napepld* in adipose tissue decreases PG and eicosanoid concentrations in adipose tissue to the same extent as the HFD treatment (Fig. 4d,e). We found in cKO-CT mice significantly increased levels of very long-chain ceramides (Fig. 4f) compared with WT-CT mice. Interestingly, we observed that HFD treatment does not affect long-chain ceramide levels, as there is no exacerbation of the cKO condition under HFD. Furthermore, cKO-CT mice exhibit a drop in the content of several phospholipids in the WAT compared with WT-CT mice (Supplementary Table 1). Altogether, these data confirm a strong impact of *Napepld* deletion on lipid metabolism in the basal state.

### *Napepld* deletion modifies inflammation and browning process

**Figure 5:**
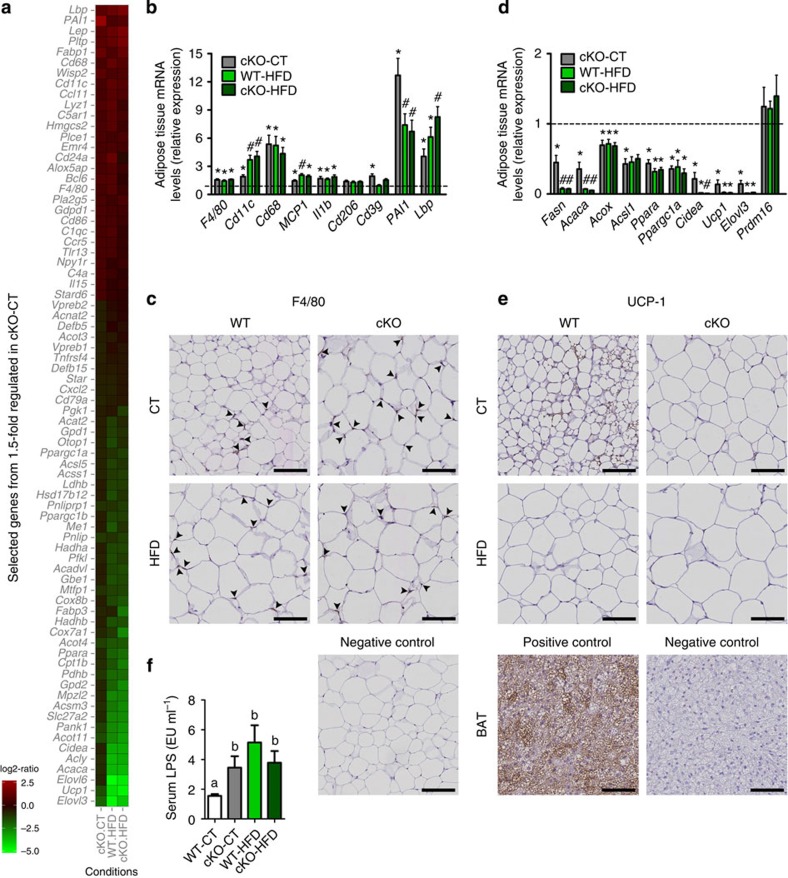
Adipose tissue *Napepld* deletion induces inflammation and altered metabolism in SAT. (**a**) Heat map from microarray analysis representing selected genes involved in inflammation or lipid metabolism, based on the list of genes down or upregulated 1.5-fold in cKO-CT versus WT-CT mice. See also Supplementary Fig. 4 and Supplementary Data 1. (**b**) mRNA expression of adipose tissue inflammation markers measured by RT–qPCR (*n*=20–27 for *F4/80*, *Cd11c*, *MCP1* and *Il1b* and *n*=6–10 for the other markers). The black-dotted line represents the WT-CT levels for mRNA expression. Data in **b** regarding inflammatory markers correspond to the results of three independent experiments. (**c**) Representative pictures of staining for F4/80 in SAT. Scale bar, 100 μm. (*n*=6–10). See also Supplementary Fig. 5. (**d**) mRNA expression of adipose tissue metabolism markers measured by RT–qPCR (*n*=6–10). The black-dotted line represents the WT-CT levels for mRNA expression. (**e**) Representative images of staining for UCP1 in SAT. Scale bar, 100 μm. (*n*=6–10). See also Supplementary Fig. 5. (**f**) Plasma LPS levels (EU per ml) measured in the portal vein (*n*=6–10). Data are presented as the mean±s.e.m. Data with different superscript letters are significantly different (*P<*0.05) according to *post-hoc* one-way analysis of variance. ‘*’ indicates a significant difference (*P<*0.05) versus WT-CT, and ‘#’ indicates a significant difference (*P<*0.05) versus WT-CT and cKO-CT according to *post-hoc* one-way analysis of variance.

To better explain the increased fat mass and altered lipid profile observed following the deletion of *Napepld* in adipose tissue, we analyzed the gene expression profiles in the WAT by microarray. These revealed an increased expression of a large number of genes involved in inflammatory pathways and a decreased expression of numerous genes involved in adipose tissue metabolism in cKO-CT, WT-HFD and cKO-HFD mice compared with WT-CT mice (Fig. 5a). Genes were assigned to relevant pathways by analyzing gene ontology using the bioinformatics tool DAVID (Supplementary Fig. 4). Figure 5a represents relative ratios in our experimental groups of selected genes that were down or upregulated by at least 1.5-fold in cKO-CT versus WT-CT mice. The entire list of genes is available in Supplementary Data 1. To confirm the results obtained from the microarray analyses, we performed quantitative PCR (qPCR) measurements on key inflammatory markers as well as on lipid metabolism and browning markers. All of the qPCR results supported the results from the microarrays and confirmed that cKO mice exhibit a markedly increased inflammatory tone in adipose tissue (Fig. 5b). It is worth noting that the levels of inflammatory markers in cKO-CT mice are similar to those of WT-HFD and cKO-HFD mice or are even more pronounced than under HFD conditions, emphasizing the effect of *Napepld* deletion on adipose tissue inflammation under physiological conditions. We have confirmed the presence of inflammation in cKO mice by histological staining for the F4/80 marker in WAT sections (Fig. 5c, quantification in Supplementary Fig. 5). With regard to lipid metabolism, we observed a decreased expression of several genes involved in lipogenesis (*Fasn* and *Acaca*), β-oxidation (*Acox1* and *Acsl1*) or key regulators of lipid metabolism (*Ppara* and *Ppargc1a*) in cKO-CT mice (Fig. 5d). Seeking a mechanistic explanation for the increased fat accumulation, we identified by microarray a marked downregulation of key markers of browning, namely *Ppargc1a*, *Ucp1*, *Cidea* and *Elovl3* in cKO-CT mice (Fig. 5a). This downregulation was confirmed by qPCR (Fig. 5d) and by immunological staining for UCP1 (Fig. 5e, quantification in Supplementary Fig. 5). Because we observed increased fat mass but no increase in food intake, these results suggest the impact of adipose *Napepld* deletion on the browning process. These different markers were similar between cKO-HFD and WT-HFD (Fig. 5b–e). We and others have linked the increased expression of lipopolysaccharide (LPS) binding protein (LBP) in the adipose tissue to inflammation and plasma LPS levels^23^,^24^. As *Lbp* mRNA were drastically increased in the adipose tissue of cKO mice according to both microarray and qPCR data, we measured plasma LPS levels, and found that the increased *Lbp* mRNA expression and the higher inflammation in cKO-CT mice were associated with a significant increase in metabolic endotoxemia (that is, increased plasma LPS levels^25^) (Fig. 5f).

### *Napepld* deletion impairs adaptation to cold exposure

**Figure 6:**
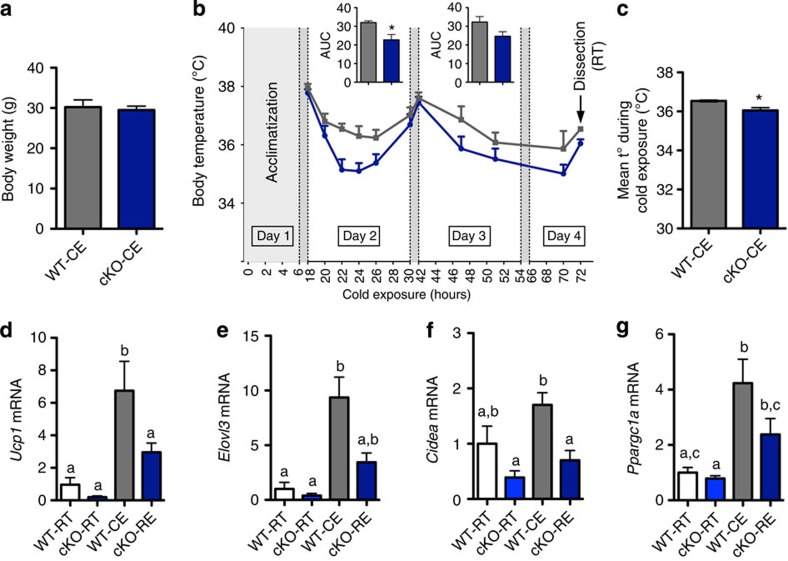
Adipose tissue *Napepld* deletion induces an altered response to cold exposure. (**a**) Total body weight (g; *n*=7). (**b**) Body temperature evolution (°C) during 72-h cold exposure (*n*=7). Inserts represent the area under the curve (AUC) of day 2 and 3, with baseline set at 34 °C. (**c**) Mean body temperature (t°) measured in °C during cold exposure (*n*=7). (**d**) *Ucp1* mRNA levels, (**e**) *Elovl3* mRNA levels, (**f**) *Cidea* mRNA levels and (**g**) *Ppargc1a* mRNA levels (relative expression) in SAT of room temperature (RT) and cold-exposed (CE) WT and cKO mice (*n*=7). Data are presented as mean±s.e.m. Two-way repeated-measure analysis of variance (ANOVA; genotype × time) revealed a significant effect of genotype (*P=*0.02), a significant effect of time (*P<*0.0001), and a non-significant interaction between factors (*P*=0.4). *Post-hoc* pairwise comparisons with Bonferroni correction did not show significant differences at any of the time points in **b**. Data with ‘*’ are significantly different (*P<*0.05) according to the unpaired two-tailed Student *t*-test. Data with different superscript letters are significantly different (*P<*0.05) according to *post-hoc* one-way analysis of variance.

To investigate whether reduced WAT browning is a causal factor for, or a metabolic consequence of, the phenotype observed, we submitted body weight-matched WT and cKO mice (Fig. 6a) to cold exposure for 72 h. Under normal conditions, this effectively upregulates *Ucp1* and consequent heat-production (‘browning’) in WT mice, in an attempt to counter the drop in environmental temperature^26^. cKO mice maintained a lower body temperature throughout cold exposure and had a significantly lower mean body temperature after 72 h of cold exposure (Fig. 6b,c). qPCR markers of the browning programme in WAT (*Ucp1, Elovl3, Cidea* and *Ppargc1a*) indicated that cold exposure resulted in normal browning of the WAT in WT mice, but that this induction was impaired in cKO mice (Fig. 6d–g).

### Browning phenotype is mainly conserved in *ex vivo* explants

Because adrenergic stimulation of WAT induces the browning process, this phenomenon *in vivo* is considered as a sympathetic event^27^. Indeed, many actions of so-called ‘browning agents’ may be traced back to indirect mechanisms that lead to activation of the sympathetic nervous system (SNS) and subsequent induction of browning^27^. However, some agents may have an impact on browning in a direct and cell-autonomous manner. To further understand the impact of adipose tissue-specific *Napepld* deletion on the browning process, we wondered if this phenotype was regulated by cell-autonomous functions or was linked to changes in the sympathetic tone *in vivo*. To address this question, we isolated subcutaneous fat pads (explants) and cultured them *ex vivo* for 24 h to eliminate peripheral adrenergic stimulations. Interestingly we found that after 24 h incubation in culture medium, adipose tissue explants from cKO mice tend to reproduce the *in vivo* phenotype on browning markers, although this decrease did not reach statistical significance (Supplementary Fig. 6). These results suggest that the effect of *Napepld* deletion on browning cannot entirely be attributed to a change in the sympathetic drive. Furthermore, we did not find any changes in mRNA expression of the β3 receptor *in vivo*, which could have been expected as β3 receptors are closely regulated at the expression level by sympathetic inputs (Supplementary Fig. 6).

### Adipose tissue *Napepld* deletion changes gut microbiota

**Figure 7:**
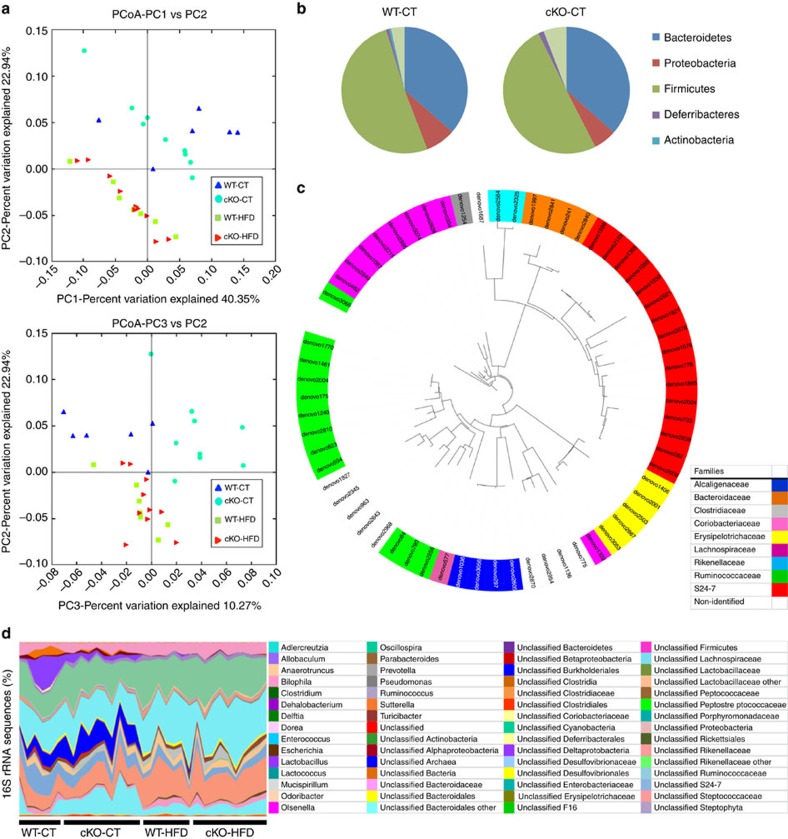
Adipose tissue *Napepld* deletion affects gut microbiota composition. (**a**) PCoA based on the weighted UniFrac analysis on operational taxonomic units (OTUs; *n*=6–10). Each symbol representing a single sample is coloured according to the group. (**b**) Composition of abundant bacterial phyla identified in the gut microbiota of WT-CT and cKO-CT mice (*n*=6–10). (**c**) The different OTUs significantly affected by adipose tissue *Napepld* deletion under CT diet. A representative 16S rRNA gene from each of the 64 differentially expressed OTUs in cKO-CT versus WT-CT mice was aligned and used to infer the phylogenetic tree presented in this figures (*n*=6–10). The colour in front of the OTU indicates the family of the OTU. (**d**) Relative abundances (percentage of 16S rRNA sequences) of the various bacterial genera in each sample among each group of mice (*n*=6–10). In **b**–**d**, the different phyla, families and genera are represented by different colour codes. See also Supplementary Tables 3–5.

The metabolic endotoxemia observed in cKO-CT mice indicates a putative shift in gut microbiota composition. Because we previously demonstrated a strong association between adipose tissue, eCB content and gut microbiota^14^,^15^, we analyzed the gut microbiota using high-throughput sequencing. Consistent with previous studies^24^,^25^,^28^, we observed a significant change in gut microbiota composition under HFD compared with the CT diet. Interestingly, the deletion of *Napepld* profoundly affected gut microbiota under CT diet conditions, as observed in the Principal coordinates analysis (Fig. 7a). More specifically, the abundance of 64 operational taxonomic units is significantly different in cKO-CT mice compared with WT-CT mice (Fig. 7c). At the taxonomic level, two phyla, six families and eight genera were significantly modified in cKO-CT mice compared with WT-CT mice (Fig. 7b–d and Supplementary Tables 3–5). Interestingly, gut microbiota from cKO-CT mice differed from that of the WT-CT mice but also from that of the HFD-treated mice (Fig. 7a,d), suggesting that the effects of *Napepld* deletion on gut microbiota may be different than those induced by the HFD treatment. This finding strongly suggests that the deletion of *Napepld* in adipose tissue has a profound influence on gut microbiota composition in physiological conditions and thereby suggests for the first time the existence of an adipose tissue to gut microbiota axis.

### Long-term antibiotic treatment improves glucose homeostasis

**Figure 8:**
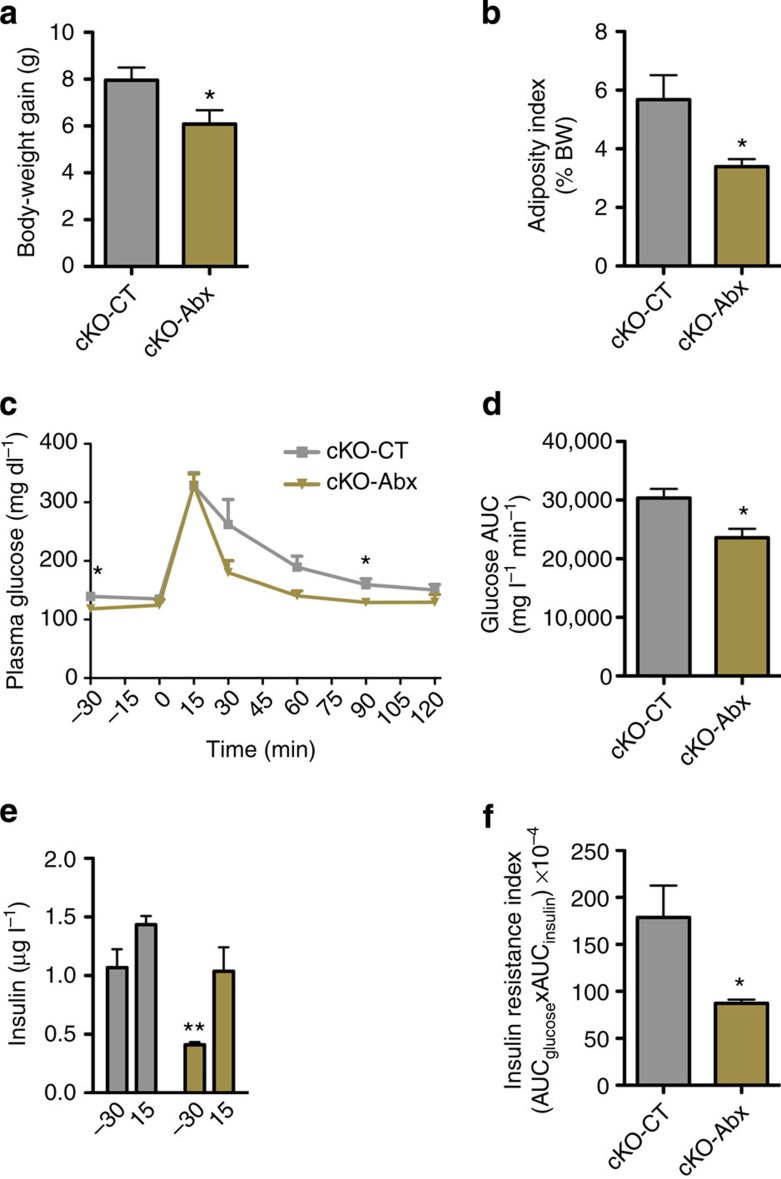
Antibiotic treatment improves metabolism in adipose tissue *Napepld*-deleted mice. (**a**) Total body-weight gain (g; *n*=5). (**b**) Adiposity index (% Total body weight) after long-term antibiotic treatment (*n*=5). (**c**) Plasma glucose (mg dl^−1^) profile and (**d**) the mean area under the curve (AUC) measured between 0 and 120 min after glucose loading (mg l^−1^ min^−1^; *n*=5). (**e**) Plasma insulin levels at 30 min before and 15 min after glucose loading (μg l^−1^) (*n*=5). (**f**) Insulin resistance index determined by multiplying the AUC of blood glucose by the AUC of insulin. Data are presented as mean±s.e.m. Data with ‘*’ are significantly different (*P<*0.05) according to the unpaired two-tailed Student *t*-test, or following a two-way statistical analysis in **c**. Data with ‘**’ indicates a significant difference (*P<*0.01) versus time point −30 min in cKO mice in **e**.

To investigate if gut microbiota may influence the phenotype observed following *Napepld* deletion, we treated mice with antibiotics for 12 weeks. Interestingly we found that long-term antibiotic treatment in cKO mice under CT (cKO-Abx) reduced body-weight gain and fat mass development (Fig. 8a,b). Antibiotics also improved glucose tolerance and insulin resistance index in cKO mice (Fig. 8c–f), suggesting a direct impact of gut microbiota on energy and glucose homeostasis.

### Gut microbiota transfer partially replicates the phenotype

**Figure 9:**
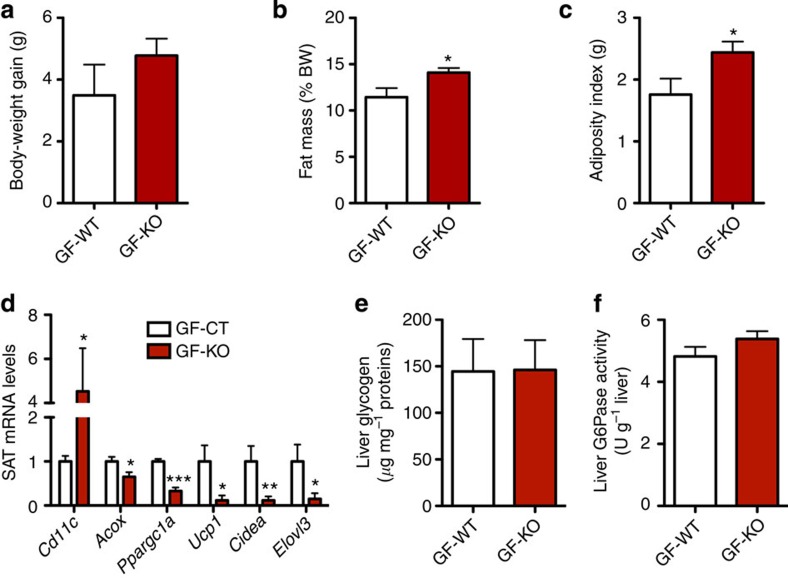
Gut microbiota transfer to germ-free (GF) mice reproduces part of the phenotype. (**a**) Body-weight gain (g) after gut microbiota transfer to germ-free mice (*n*=7). (**b**) Fat mass weight in percentage of body weight after gut microbiota transfer to germ-free mice (%; *n*=7). (**c**) Adiposity index (g) after gut microbiota transfer to germ-free mice (*n*=7). (**d**) mRNA expression of *Cd11c*, *Acox*, *Ppargc1a*, *Ucp1*, *Cidea* and *Elovl3* in SAT (*n*=7). (**e**) Liver glycogen content (μg per mg proteins; *n*=7). (**f**) Liver G6Pase activity (U per g liver; *n*=7). Data are presented as mean±s.e.m. Data with *P<0.05, **P<0.01 and ***P<0.001 are significantly different according to the unpaired two-tailed Student’s *t*-test.

To see whether the altered gut microbiota composition in cKO mice is a causal factor for, or a metabolic consequence of, the phenotype observed and to further elucidate whether the profound shift in gut microbiota composition observed in cKO mice may contribute to the phenotype, we transferred gut microbiota from body-weight-matched WT or cKO mice into germ-free (GF) recipient mice. Both donors and recipients were kept on a CT diet. After 4 weeks, we found that gut microbiota transfer from cKO donors significantly increased fat mass gain and the adiposity index (sum of the weights of the different adipose tissue depots) (Fig. 9b,c). There was a trend towards increased total body-weight gain but this did not reach statistical significance (Fig. 9a). We found that gut microbes from cKO mice increase adipose tissue inflammation (increased *Cd11c* mRNA levels—Fig. 9d) and markedly decrease markers of β-oxidation and browning (*Acox*, *Ppargc1a*, *Cidea*, *Elovl3*, *Ucp1*—Fig. 9d). To investigate if gut microbiota transfer may also affect insulin sensitivity, we analyzed *Slc2a4* (GLUT4) expression in subcutaneous adipose tissue (SAT) and muscle and glucose-6-Phosphatase (*G6pc*) expression in the liver and found that the microbiota transfer from cKO mice did not affect these parameters. Furthermore, microbiota transfer did neither alter liver glycogen levels nor G6Pase activity (Fig. 9e,f). Thus, these results suggest that gut microbiota transfer is sufficient to rapidly reproduce the phenotype observed in adipose tissue of *Napepld*-deleted mice (that is, fat gain and browning), whereas the impact on glucose metabolism is not yet observed. Together, the results obtained following antibiotic exposure and microbiota transfer confirm the key role played by gut microbiota in shaping the host phenotype and demonstrate the critical influence of adipose tissue NAPE-PLD on gut microbiota.

## Discussion

**Figure 10:**
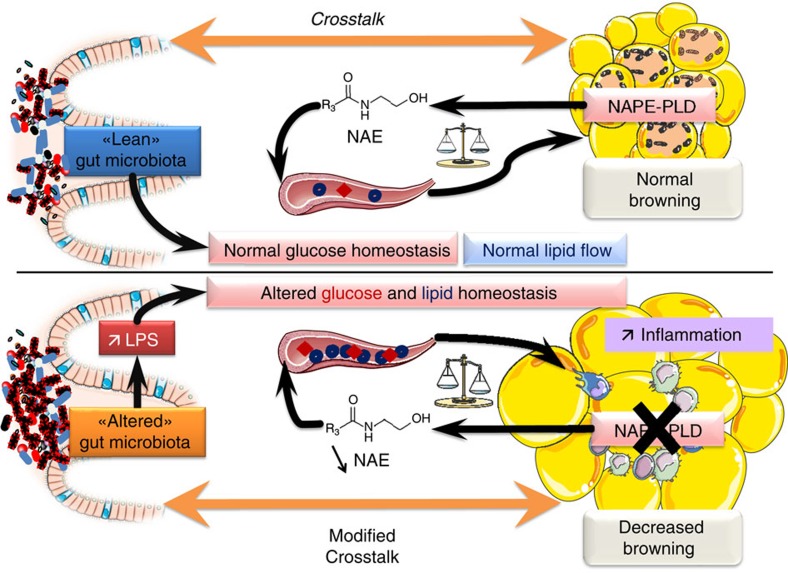
Schematic overview of the phenotype observed following *Napepld* deletion in adipocytes. When NAPE-PLD is present and functional, adipose tissue homeostasis is maintained and this participates in a normal crosstalk between adipose tissue and gut microbiota. Moreover the presence of NAPE-PLD participates in normal glucose homeostasis and lipid flows. When NAPE-PLD is absent, adipose tissue metabolism is deregulated, with an increase in inflammation, decrease in the browning capacity and excessive fat mass development. *Napepld* deletion also induces alterations in glucose homeostasis and alters normal lipid flows and lipid homeostasis in circulation and in adipose tissue. Altered adipose metabolism following *Napepld* deletion induces a dysbiosis of the gut microbiota, which in turn participates in the metabolic alterations observed in the adipose tissue.

The ECS and its related bioactive lipids such as NAEs play key roles in the regulation of energy homeostasis. In this paper, we discovered the essential role of the eCB synthesizing enzyme NAPE-PLD in adipose tissue (summarized in Fig. 10). Mice lacking the *Napepld* gene in their adipose tissue are prone to obesity and associated metabolic disorders. Remarkably, the phenotype of cKO mice develops in a physiological state (that is, CT diet). This indicates that NAPE-PLD plays an important role in the regulation of basal metabolism, energy homeostasis and inflammation. Surprisingly, *Napepld*-deleted mice under HFD have higher body-weight gain and insulin resistance index, whereas other metabolic parameters are not exacerbated under this pathological condition. Since the levels of adipose tissue NAEs are similar between WT and cKO mice under HFD, this may explain why we do not observe increased inflammation and altered lipid metabolism under HFD. Nevertheless, *Napepld* deletion is effective when differentiation of adipose tissue is complete, thus before the beginning of HFD treatment. Therefore increased body-weight gain and insulin resistance may be directly attributed to *Napepld* deletion whereas other metabolic alterations are probably due to long-term NAEs reduction.

Using different approaches (that is, microarray, qPCR and histology), we determined that cKO mice develop a marked inflammatory tone. This phenotype may be explained by different mechanisms, including the fact that *Napepld* deletion decreased the levels of the anti-inflammatory PEA. Indeed, PEA has been identified previously as a bioactive lipid with anti-inflammatory properties^9^,^10^,^29^. Moreover, the altered regulation of several PGs, phospholipids, ceramides and eicosanoids may contribute to altering the regulation of inflammatory pathways^30^. Interestingly, it has been shown that an inflammatory stimulus such as LPS reduces *Napepld* expression and PEA production in RAW264.7 macrophages^31^. These *in vitro* data, together with our *in vivo* findings, support a role for NAPE-PLD in regulating the normal inflammatory response.

The ECS plays an important role in regulating glucose homeostasis^32^,^33^,^34^. Accordingly, our findings highlight the impact of adipose tissue *Napepld* on glucose metabolism. Perturbations of glucose homeostasis have been linked to adipose tissue inflammation^35^,^36^, suggesting that the inflammatory tone developed in our model contributes to the observed glucose intolerance and insulin resistance. We also found that insulin resistance occurs mainly in the liver rather than skeletal muscle or adipose tissue. Nevertheless, we cannot exclude that insulin resistance could affect those organs at a later time since phosphorylation of IRβ is also significantly affected in skeletal muscle and there is an important trend towards reduced insulin-induced phosphorylation of IRβ in adipose tissue. Thus, the specific kinetic of development of insulin resistance warrants further investigations.

In addition, we found that *Napepld* deletion in adipose tissue leads to an increase in circulating TAG and cholesterol levels, underlying perturbations in lipid metabolism. To further elucidate the mechanisms linking bioactive lipids produced by NAPE-PLD and other lipid candidates known to play a major role in inflammation and insulin resistance, we performed a thorough lipidomic analysis, including ceramides, eicosanoids and PGs. Ceramides link inflammation to insulin resistance^37^. Moreover, a recent study reported a direct link among long-chain ceramides, eCB and insulin action in the liver^38^. Interestingly we observed that cKO mice exhibit increased levels of long-chain ceramides in their adipose tissue, suggesting that altered NAE production in adipose tissue may impact ceramide levels and subsequently lead to other metabolic disturbances. The eCB and eicosanoid metabolisms are closely related^17^,^39^. Eicosanoids and PG are synthesized from arachidonic acid (AA) via the COX pathway, which can also metabolize eCB^39^. Moreover, in cKO-CT mice, we found an upregulation for several genes involved in the regulation of inflammation and immunity as well as bioactive lipid metabolism (*Alox5ap, Pla2g5* and *Plce1*; Fig. 5a). In contrast, we observed a decrease in eicosanoids and PG in the adipose tissue of cKO mice as well as in the adipose tissue of HFD-treated mice, where an increase in these pro-inflammatory lipid mediators could have been expected^22^. However, recent data have suggested that these lipids may exhibit both pro- and anti-inflammatory properties according to the pathological situation. On the other hand, PG derivatives can be synthesized by the metabolism of eCB via the COX2 pathway, leading to the formation of PG-glycerol esters or PG-ethanolamides, which exert anti-inflammatory effects^40^,^41^. In addition, these bioactive lipids may act as resolvins, thereby contributing to the complex resolution of inflammation^30^. Whether the decrease in PGD_2_ and PGE_2_ observed here contributes to the modulation of these complex interactions requires further investigation. Finally, AA levels are unchanged in cKO-CT mice and are decreased under HFD compared with WT-CT mice (Supplementary Fig. 7). The global altered lipid profile observed in our model suggests that the decreased level of the substrate (AA) may explain why PGs are not increased. Taken together, these data shed light on the influence of NAPE-PLD on bioactive lipid levels in adipose tissue and the pronounced altered adipose tissue lipid metabolism in our mice model. Furthermore, we found increased levels of NAPEs (NAEs precursors, Supplementary Fig. 2) in adipose tissue of cKO mice compared with WT mice, confirming data obtained in previous studies^4^,^11^,^21^. These lipids could also be potential contributors to the phenotype observed since some NAPEs have been shown to present metabolic properties, namely on energy homeostasis^42^. Nevertheless, this statement warrants further investigation in our model.

cKO mice also exhibited decreased mRNA levels of *Ucp1* and other brown fat cell-enriched genes, such as *Cidea* and *Ppargc1a*, in their WAT, suggesting that their adipose tissue loses thermogenic potential. This finding was further supported by the microarray analysis, which indicated a reduction in the expression of several genes generally associated with brown/beige adipocytes, such as Eva1 (also known as myelin protein zero-like 2, *Mpzl2*) and the mitochondrial genes *Cox7a1* and *Cox8b*. It has recently been suggested that a subset of adipocytes in the SAT deposits can activate a thermogenic programme, namely beige or brite cells^43^,^44^. Animals deficient in functional beige cells develop obesity, in conjunction with a huge increase in SAT deposits^45^. Remarkably, we observed that more than 10 genes involved in the regulation of these metabolic processes were significantly affected in the absence of NAPE-PLD. Based on these observations, we propose that the increased fat mass development is associated with an altered browning process in the adipose tissue and a reduced capacity to develop a normal thermogenic programme. Interestingly, the microarray analysis also revealed the downregulation of *Fabp3* and mitochondrial fatty acid oxidation genes, such as *Acss1*, *Acsl5* and *Cpt1b*. These enzymes have been found to be upregulated after cold exposure or β-adrenergic stimulation and are linked to a brown-like transformation of the WAT^46^. Their downregulation might reflect reduced β-oxidation due to the further loss of the BAT-characteristics of the WAT. This defect in β-oxidation may potentially contribute to the elevated levels of circulating lipids observed. Moreover, pharmacological blockade of the CB_1_ receptor induces the activation of BAT thermogenesis associated with enhanced glucose and lipid utilization^47^,^48^, while OEA has been recently implicated in the enhancement of β-adrenergic-mediated thermogenesis in rats^49^, linking ECS and thermogenesis. Altogether, the altered lipid metabolism observed in our model could be linked to the altered browning process. For example, PGE_2_ has recently been identified as a key regulator of white-to-brown adipogenesis^50^ and the reduced PGE_2_ levels observed in the WAT may therefore participate in decreased browning. To further explore the effects of *Napepld* deletion on the browning process, we exposed cKO and WT mice to cold exposure and found that *Napepld*-deleted mice clearly present an altered induction of cold-induced browning process. This process seems to be due to *Napepld* deletion independently of a sympathetic drive since adipose tissue explants from cKO donors tend to develop the same phenotype. Overall, these data clearly indicate that adipose tissue NAPE-PLD is a key enzyme involved in the regulation of energy homeostasis by regulating the browning process. Nevertheless, the causal impact of *Napepld* deletion on browning process merits further investigations to ascertain the direct effects on browning, independently of a heat-loss mechanism or sympathetic nervous system activation^27^.

Using 454-pyrosequencing, we discovered that altering the NAE synthesis in adipose tissue profoundly alters the gut microbiota composition. For instance, the genera *Lactobacillus* and *Allobaculum* were decreased in cKO-CT mice compared with WT-CT mice (Supplementary Table 5). We have previously reported that HFD feeding reduces the abundance of *Allobaculum*, whereas prebiotics increase this genus; reduce fat mass, metabolic inflammation and *Lbp* mRNA expression; and increase insulin sensitivity^24^. Similarly, the abundance of *Allobaculum* is increased in rats that were fed with berberine, which prevents obesity and insulin resistance on HFD treatment^51^. Several strains of *Lactobacillu*s are commonly used probiotics^52^,^53^. It is therefore not unconceivable that the effect of *Napepld* deletion in adipose tissue induces metabolic alterations associated with metabolic functions assumed by gut microbes. We also observed a significant impact of HFD treatment on the gut microbiota composition, confirming previously published data^24^. Furthermore, *Napepld* deletion leads to an increase in portal LPS, suggesting an altered intestinal barrier function^54^. We thus postulate that the decreased production of NAEs in the adipose tissue has an impact on the gut microbiota and the gut barrier function^14^.

By treating cKO mice with antibiotics and by transferring gut microbes from cKO and WT mice to GF recipients, we could demonstrate the contribution of the gut microbiota on the phenotype observed in cKO mice. These results acknowledge an important role for the gut microbiota, which seems to be independent of body weight, as adipocyte-specific *Napepld* deletion results in a distinct gut microbiota composition from that obtained under HFD conditions and as transfer of gut microbiota from body-weight-matched donors is sufficient to effectively transfer part of the phenotype. However, we may not completely rule out the complementary association of body weight and gut microbiota modulation over the long term since antibiotic treatment reduces body weight and improves glucose homeostasis in cKO mice. To note, only 4 weeks follow-up after gut microbiota transfer (compared with 8 weeks in the other *in vivo* studies) were enough to partly reproduce the phenotype. Conversely, insulin sensitivity and glucose tolerance may not yet be affected 4 weeks after gut microbiota transfer in our mice model, but rather observed after a long-term modulation of gut microbiota (that is, antibiotic treatment). Also, whether one or several specific gut microbes contribute to this phenotype requires further investigations.

In conclusion, our study highlights the essential contribution of the adipose tissue NAE synthesis pathway, to whole-body energy metabolism and physiology. In the absence of this functional synthesis pathway in the basal state, mice develop obesity, adipose tissue inflammation, insulin resistance, glucose intolerance and perturbation of the lipid metabolism. This phenotype is partly mediated by the alteration of gut microbiota composition and by an altered browning programme (Fig. 10). Taken together, these data underlie the importance of tissue-specific differences in ECS regulation, with special emphasis on adipose tissue.

## Methods

### Mice

*Generation of adipose tissue Napepld cKO mice*. Adipose tissue-specific *Napepld*-deleted mice (cKO mice) were generated by crossing mice bearing the *Cre* recombinase expressed under the control of the *Fabp4* promoter (*Fabp4-Cre*) (C57BL/6 background, Jackson-Laboratory, Bar Harbor, ME, USA) with mice harbouring a *loxP*-flanked *Napepld* allele. *Napepld* loxed mice were generated as previously described^55^. Deletion was effective when adipose tissue reaches maturity and mice were born at normal Mendelian ratios.

All mouse experiments were approved by and performed in accordance with the guidelines of the local ethics committee for animal care of the Health Sector of the UCL under the supervision of Prof. F. Lemaigre and Prof. JP Dehoux and under the specific number 2010/UCL/MD/022. Housing conditions were specified by the Belgian Law of 29 May 2013 regarding the protection of laboratory animals (agreement number LA1230314).

*cKO experiments*. Cohorts of 8-week-old male cKO mice and WT littermates were housed in groups of two mice per cage (filter-top cages) with free access to food and water. The mice were fed a CT (AIN93Mi, Research Diet) or a HFD (60% fat, D12492, Research Diet). Treatment continued for 8 weeks. This experiment was replicated independently three times. The control mice were WT littermates harbouring the *Napepld loxP*-flanked allele but not the *Cre* recombinase. Body weight, food intake and water consumption were recorded once a week. Body composition was assessed once a week using 7.5-MHz time-domain NMR (TD-NMR) (LF50 Minispec, Bruker, Rheinstetten, Germany).

After 7 weeks of treatment, an OGTT was performed as previously described in freely moving mice^25^,^32^. To analyze the insulin signalling pathway, mice received 5 U insulin (Actrapid; Novo Nordisk A/S, Denmark) under anaesthesia (isoflurane, Forene, Abbott, Queenborough, Kent, England), or an equal volume of PBS into the portal vein to analyze signalling response to insulin. Three minutes after injection, mice were killed and liver, SAT and gastrocnemius skeletal muscle were rapidly dissected.

At the end of the treatments, the mice were anaesthetized with isoflurane after a 6-h fasting period. Portal and cava vein blood samples were collected for further analysis. After exsanguination, mice were killed by cervical dislocation. Tissues were precisely dissected, weighed and immediately snap-frozen in N_2_ and stored at −80 °C for further analysis.

*Cold exposure experiment*. Cohorts of 8-week-old male cKO mice and WT littermates were reared at room temperature under standard housing conditions (filter-top cages) with free access to food and water for 10 weeks. The mice were fed a CT diet (AIN93Mi, Research Diet, New Brunswick, NJ, USA). Body weight, food intake and water consumption were recorded once a week. Body composition was assessed once a week using 7.5-MHz TD-NMR. After 10 weeks follow-up, two groups of age- and body weight-matched cKO and WT mice were separated and individually housed. One half of the animals from each genotype was transferred to a cold room at 8 °C, whereas the other half remained at room temperature (*n*=7–9/group). Mice were fasted during the day period and fed *ad libitum* during the night. Mice were kept in the cold room for 72 h and after 18 h acclimation in the cold room, body temperature was monitored at different intervals during two days with a rectal probe (RET-3, World Precision Instruments, Aston Stevenage, UK). After 72 h, all mice were sacrificed as described above.

*Antibiotic treatment experiment*. Cohorts of 8-week-old C57/Bl6 female cKO mice were housed in groups of 2 or 3 mice/cage (filter-top cages) with free access to food and water. The mice were fed a CT diet for 12 weeks. Half of the mice (*n*=5 per group) received antibiotics (1.0 g l^−1^ ampicillin (Sigma, St Louis, MO) and 0.5 g l^−1^ neomycin (Sigma)) in their drinking water during the experimental period^14^. Body weight was recorded once a week. Body composition was assessed once a week using 7.5-MHz TD-NMR. After 11 weeks, an OGTT was performed as described above. After 12 weeks follow-up, mice were killed as described above.

*Gut microbiota transplantation experiments*. The caecal contents of 3 cKO mice and 3 WT littermates (body weight matched) were transplanted into 14 GF mice (7-week-old Swiss-Webster males, Taconic, Hudson, NY, USA) as previously described^56^. Each donor was used to transplant two or three GF recipients. Each caecal sample (150 mg) was sampled in an anaerobic chamber and suspended in PBS (1.5 ml per caecum). Caecal contents were then immediately administered (0.15 ml per mouse) to the GF mice. GF mice were housed individually in ventilated cages (IVC AERO GM500, Tecnilab-BMI, Someren, The Netherlands) and fed a CT diet for 4 weeks. Body weight, food intake and water consumption were recorded once a week. Body composition was assessed once a week using 7.5-MHz TD-NMR. After 4 weeks follow-up, mice were killed as described above.

### Insulin resistance index

The plasma insulin concentrations were measured in plasma collected from tail blood during OGTT using an ELISA Kit (Mercodia, Uppsala, Sweden) according to the manufacturer’s instructions. The insulin resistance index was determined by multiplying the area under the curve of both the blood glucose (−30 to 120 min) and the plasma insulin (−30 to 15 min) obtained from the OGTT.

### RNA extraction and real-time qPCR analysis

Total RNA was prepared from tissues using the TriPure reagent (Roche). The quantification and integrity analysis of the total RNA were performed by running 1 μl of each sample on an Agilent 2100 Bioanalyzer (Agilent RNA 6000 Nano Kit, Agilent). The complementary DNA was prepared by reverse transcription, and real-time qPCR was performed as previously described^24^. *RPL19* RNA was chosen as the housekeeping gene. Primer sequences are provided in the Supplementary Table 2.

### Microarray analysis

Equal amounts of RNA from five mice per group were pooled within each group. Microarrays were performed as previously described^57^. Mouse gene ST microarray chips were used for hybridization (MoGene 1.0 ST, Affymetrix). The WT expression kit (Ambion) was used for complementary RNA preparation from the total RNA. The hybridization, wash and scan were done according to the Affymetrix kits and procedures specific to the mouse gene ST chips. After the scan, the quality controls of the hybridization were checked using the Affymetrix Gene Expression Console software. Using the Affymetrix APT suite tools, we normalized the data by the RMA-Sketch procedure and computed the signal detection *P* values using the DABG algorithm. All the probe sets that have the DABG *P* value >0.05 in all conditions were discarded from the analysis. The rest of probes sets were kept for fold-change analysis. Functional annotation and pathway analysis was done using the DAVID web tool^58^. Both tools were fed with the list of selected official gene names as input, and the threshold of significance was set by default to *P* values <0.05.

### DNA isolation from mouse caecal samples and qPCR and sequencing

Caecal contents were collected and kept frozen at −80 °C until use. Metagenomic DNA was extracted from the caecal content using the QIAamp DNA Stool mini-kit (Qiagen, Hilden, Germany) according to the manufacturer’s instructions. The V1-V3 region of the bacterial 16S rRNA gene was amplified using barcoded primers 27f and 534r (ref. 59), and the high-throughput sequencing results of the purified amplicons were analyzed on a Roche FLX Genome Sequencer using titanium chemistry (DNAVision, Gosselies, Belgium). The resulting reads were processed through the QIIME v1.7.0 pipeline^60^. The abundance of identified and unclassified taxa was transformed using the Hellinger method after removing taxa representing <0.01% of the total abundance. Principal coordinates analysis was calculated using the weighted UniFrac distance. Operational taxonomic units were identified using the uclust consensus taxonomy classifier with a 0.97 threshold against the Greengenes database. Phylogenetic trees were generated using QIIME 1.7.0 and visualized using iTOL v2.2.2.

### SDS–PAGE and immunoblotting

For the total lysates, tissues were homogenized with TissueLyser II (Qiagen) in RIPA buffer^61^ supplemented with a cocktail of protease inhibitors (Sigma) and phosphatase inhibitors. Equal amounts of proteins were separated by SDS–PAGE and transferred to nitrocellulose membranes. For detection of proteins of the insulin pathway, tissues were homogenized in ERK buffer (Triton X-100 0.1%, HEPES 50 mM, NaCl 5 M, Glycerol 10%, MgCl_2_ 1.5 mM and DTT 1 mM) supplemented with a cocktail of protease inhibitors and phosphatase inhibitors. Membranes were incubated overnight at 4 °C with the following antibodies diluted in Tris-buffered saline tween-20 containing 1% non-fat dry milk: NAPE-PLD (1:200; ab95397, Abcam, Cambridge, MA, USA), p-IRβ (1:1,000; sc-25103, Santa Cruz, CA, USA), p-Akt^Thr308^ (1:1,000; #2965L, Cell Signaling, Danvers, MA, USA) and p-Akt^Ser473^ (1:1,000; #4060L, Cell Signaling). Quantification of phospho-proteins was performed on six animals with insulin injection per group. The loading control was β-actin (1:10,000; ab6276) or β-tubulin for skeletal muscle (1:800; sc-9104). Full unedited blots are available in Supplementary Fig. 8.

### Histological analysis and immunohistochemistry

The tissues were fixed in 4% formaldehyde. Haematoxylin and eosin staining was performed using standard protocols on 5-μm adipose tissue sections. Adipocyte size (haematoxylin and eosin-stained sections), macrophage infiltration (F4/80: ab6640, Abcam) and UCP1 staining (ab23841, Abcam) were determined using ImageJ (version 1.48r, National Institutes of Health, Bethesda, Maryland, USA).

### Separation of adipocytes and the SVF

About 150–300 mg of SAT deposit were dissected and cut in small pieces and digested with collagenase A (Roche) for 15 min at 37 °C. Digested tissue was filtered and centrifuged at 400 g for 1 min. The infranatant containing the SVF and the supernatant containing adipocytes were washed three times in a Krebs-BSA1% solution and stored at −80 °C in Tripure Reagent (Roche) for further RNA extraction.

### Primary peritoneal macrophage isolation

Murine peritoneal macrophages were obtained by eliciting an acute peripheral inflammatory reaction with an i.p. injection of thioglycolate^62^. Isolated primary macrophages were incubated at 37 °C for 4 h, washed with PBS and then frozen in Tripure Reagent for further RNA extraction.

### Adipose tissue explants culture

Subcutaneous adipose depots from 20 mice (10 WT mice and 10 cKO mice) were precisely dissected, and all visible vessels, particles and conjunctive tissues were removed. The fat tissue was then cut into small pieces (4 mm^3^), pooled per genotype and placed into Krebs buffer (pH 7.4) containing 0.5% (w/v) fatty acid-free BSA, penicillin/streptomycin (1:100) and fungizone (1:100) (Invitrogen). A total of 200 mg adipose tissue was rinsed in PBS and incubated in 100-mm Petri dishes containing 10 ml αMEM (Invitrogen) supplemented with 0.5% (w/v) fatty acid-free BSA, penicillin/streptomycin (1:100) and fungizone (1:100). All conditions were repeated in six different dishes. Dishes were cultured for 24 h at 37 °C in a 5% CO_2_ atmosphere. The basal concentration of glucose in fresh media was 5 mmol l^−1^. At the end of the experiment, adipose material was collected and immediately frozen in liquid nitrogen, and stored at −80 °C until subsequent mRNA analysis.

### Biochemical analyses

Circulating leptin was determined using a multiplex immunoassay kit (Merck Millipore, Brussels, Belgium) and measured using Luminex technology (Bioplex, Bio-Rad, Belgium) following the manufacturer’s instructions.

For liver G6Pase activity, liver tissue was homogenized in lysis buffer (HEPES 20 mM, sucrose 250 mM, KCl 10 mM, MgCl_2_ 1.5 mM, EDTA 1 mM and DTT 1 mM) and sonicated to release membrane-bound G6Pase. Homogenates were then separately incubated with 20 mM G6P (Sigma) or 20 mM β-glycerophosphate (Sigma) to measure nonspecific phosphatase activity. Inorganic phosphate was assayed in each condition at time 0 and 10 min incubation at 37 °C. G6Pase activity was determined by calculating specific G6Pase–phosphate release as previously described^63^.

For the measurement of liver glycogen content, liver was digested in NaOH (1 M) and digestion was stopped with HCl (1 M). Digested liver was diluted in sodium acetate (1:4 vol/vol) unsupplemented or supplemented with amyloglucosidase (50 U ml^−1^; Merck Millipore) and incubated at 55 °C for 1 h to transform glycogen into glucose. Released glucose was quantified using Glucose God FS (Diasys Diagnostic and Systems, Holzheim, Germany) according to the manufacturer’s instructions.

Plasma non-esterified fatty acids, cholesterol and triglyceride concentrations were measured using kits coupling an enzymatic reaction with spectrophotometric detection of the reaction end-products (Diasys Diagnostic and Systems) according to the manufacturer’s instructions.

The portal plasma LPS concentration was measured using Endosafe-MCS (Charles River Laboratories, Lyon, France) as previously described^64^.

### Lipidomics analysis

Lipidomics were performed in collaboration with Biocrates (Innsbruck, Austria). The most biologically abundant phospholipids, ceramides and eicosanoids were quantitatively analyzed by a high-throughput flow injection electrospray ionization-tandem mass spectrometry (ESI-MS/MS) screening method or by HPLC-MS/MS(LC-MS/MS).

### Measurement of eCB and NAPEs levels

Tissues were homogenized in CHCl_3_ (10 ml), then MeOH (5 ml), H_2_O (2.5 ml) and HCl 2 N were added and the lipids extracted by vigorous mixing. The organic layer was recovered and dried under N_2_. The resulting lipid fraction was pre-purified by solid-phase extraction over silica, and NAPEs were eluted with CHCl_3_-MeOH (6:4, v/v). The resulting lipid fraction was analyzed by HPLC-MS using a LTQ Orbitrap mass spectrometer (Thermo Fisher Scientific, Waltham, MA, USA) coupled to an Accela HPLC system (Thermo Fisher Scientific). Analyte separation was achieved by using a C-18 Kinetex C-18 column (5 μm, 4.6 × 150 mm; Phenomenex, Utrecht, Netherlands) and a C-18 pre-column. Mobile phases A and B were composed of MeOH-H_2_O-NH_4_OH (75:25:0.1, v/v/v) and MeOH-NH_4_OH (100:0.1, v/v), respectively. The gradient (0.5 ml min^−1^) was as follows: from 100% A to 100% B in 15 min, followed by 10 min at 100% B and subsequent re-equilibration at 100% A. Mass spectrometry analysis in the negative mode was performed with an ESI source. The measurement of eCB were generated as previously described^32^, and the data were normalized to tissue sample weight.

### Statistical analyses

The data are expressed as the mean±s.e.m. Differences between the groups were assessed using one-way analysis of variance (ANOVA), followed by the Tukey *post-hoc* test. A two-way ANOVA analysis with a Bonferonni post-test on repeated measurements was performed for the evolution of body weight, fat mass and glycaemia during the OGTT. The data were analyzed using GraphPad Prism version 5.00 for Windows (GraphPad Software, San Diego, CA, USA). Data with different superscript letters or symbols are significantly different at *P<*0.05 according to the *post-hoc* ANOVA statistical analysis. Comparisons between the WT-CE and cKO-CE, the cKO and cKO-Abx groups, and the GF-WT and the GF-KO groups were performed using a two-tailed Student’s *t*-test. The results were considered statistically significant when *P<*0.05.

## Author contributions

P.D.C. conceived, supervised the project and designed experiments, performed experiments and contributed in the interpretation of all the results. S.L. generated *Napepld* floxed mice. L.G. contributed to the design of the experiments, performed most of the experiments, analyzed and interpreted the results. L.G., M.V.H., Ah.E. and S.M. contributed to generate figures and tables; A.E., M.V.H., Ah.E., T.D., S.M., H.P., J.C. and R.G.P.D. performed the experiments. M.B. contributed to the antibiotic treatment. C.D. contributed to isolate SVF. M.A. helped with the isolation of peritoneal macrophages. G.G.M. measured the endocannabinoids and NAPEs. N.M.D., J.-B.D. and S.L. provided reagents and participated in the discussions. L.G. and P.D.C. wrote the manuscript. All authors discussed the results, reviewed and approved the manuscript.

## Additional information

**Accession codes:** Microarray data have been deposited in GEO under accession code GSE56852.

**How to cite this article:** Geurts, L. *et al*. Adipose tissue NAPE-PLD controls fat mass development by altering the browning process and gut microbiota. *Nat. Commun.* 6:6495 doi: 10.1038/ncomms7495 (2015).

## Supplementary Material

Supplementary Figures and Supplementary Tables Supplementary Figures 1-8 and Supplementary Tables 1-5

Supplementary Data 1 Total list of genes 1.5-fold up-regulated or down regulated in cKO-CT mice compared to WT-CT mice.
