# Supplementary material for: Adipose tissue NAPE-PLD controls fat mass development by altering the browning process and gut microbiota
Source: Nat Commun. 2015 Mar 11;6:6495. doi: 10.1038/ncomms7495 (PMC4382707; doi:10.1038/ncomms7495)
Supplement: Supplementary Figures and Supplementary Tables — Supplementary Figures 1-8 and Supplementary Tables 1-5 [file ncomms7495-s1.pdf]

Supplementary informations

Supplementary Fig. 1

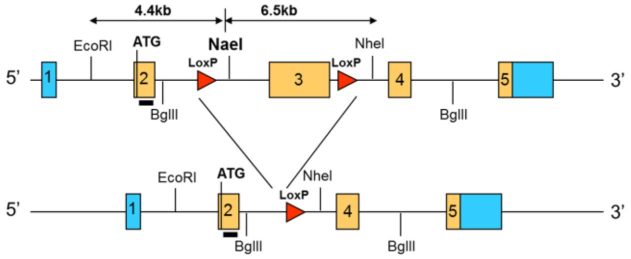

Schematic view of the *NAPE lox/lox* construction. *Napepld* $^{lox/lox}$ mice were generated by engineering loxP sites flanking exon 3, which is the most important coding exon.

Supplementary Fig. 2

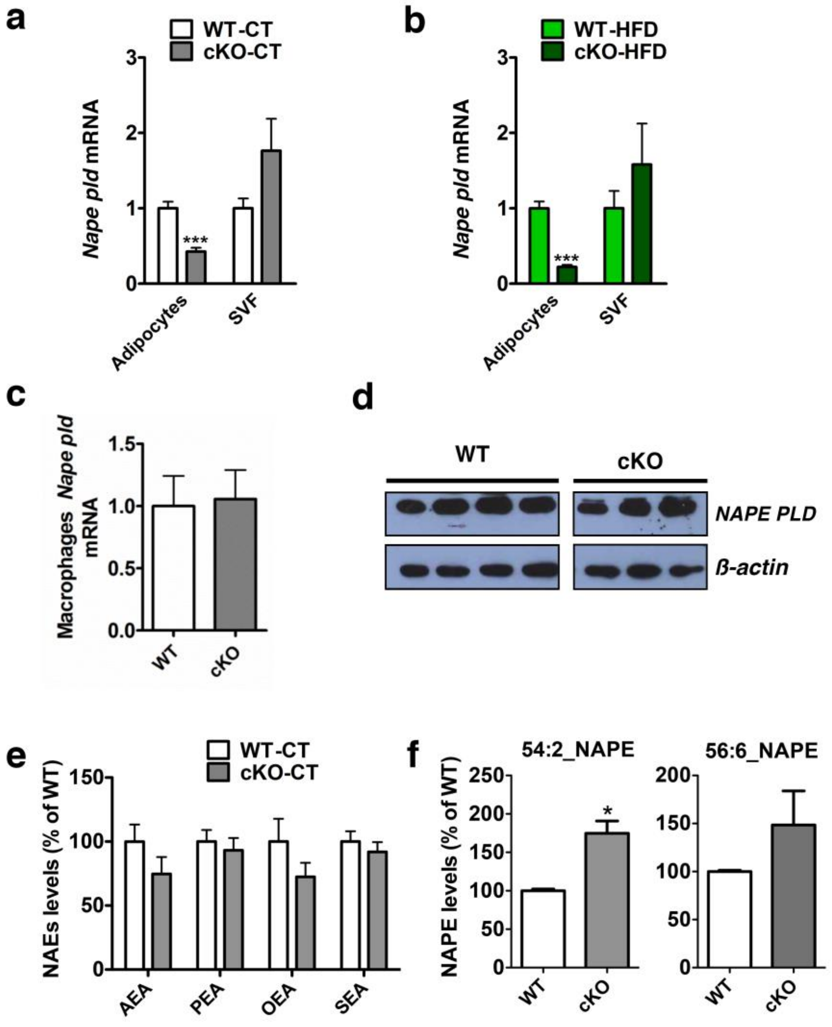

***Nape pld* deletion occurs in adipocytes but not in the stromal vascular fraction**. (**a**) mRNA expression of *Nape pld* in adipocytes and Stromal vascular fraction (SVF) separated from the SAT depot under CT diet (n=6-10). (**b**) mRNA expression of *Nape pld* in adipocytes and SVF separated from the SAT depot under HFD (n=6-10). (**c**) mRNA expression of *Nape pld* in peritoneal isolated primary macrophages. (**d**) Representative brain immunoblot of NAPE-PLD and β-actin in WT mice and cKO mice. Western blot has been performed on two independent studies on a total of n=10/group. (**e**) Brain levels of AEA, PEA, OEA and SEA (expressed as a percentage of the wild-type) measured by HPLC-MS (n=5). (**f**) SAT levels of NAPEs (54:2 and 56:6) (expressed as a percentage of the wild-type) measured by HPLC-MS (n=5). Data are shown as the means ± SEM. * indicates a significant difference ($P < 0.05$), *** indicates a significant difference ($P < 0.01$) versus WT-CT according to the unpaired two-tailed Student t-test.

Supplementary Fig. 3

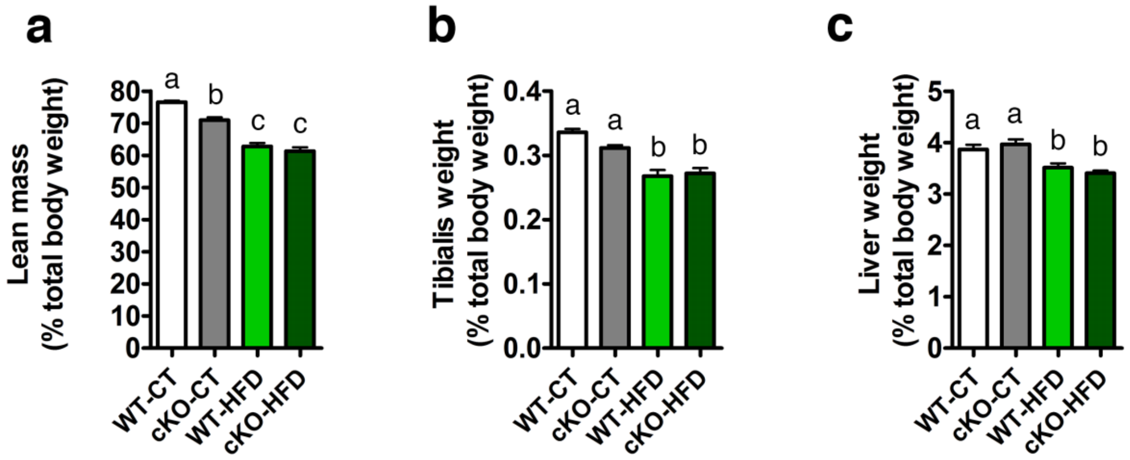

Adipose tissue *Nape pld* deletion effect on lean mass. (**a**) Total lean mass (% of total body weight) measured by TD-NMR (n=20-27). (**b**) Tibialis muscle weight (% of total body weight) (n=20-27). (**c**) Liver weight (% of total body weight) (n=20-27). Data are shown as the means ± SEM. Data with different superscript letters are significantly different ($P < 0.05$) according to post hoc ANOVA one-way.

Supplementary Fig. 4

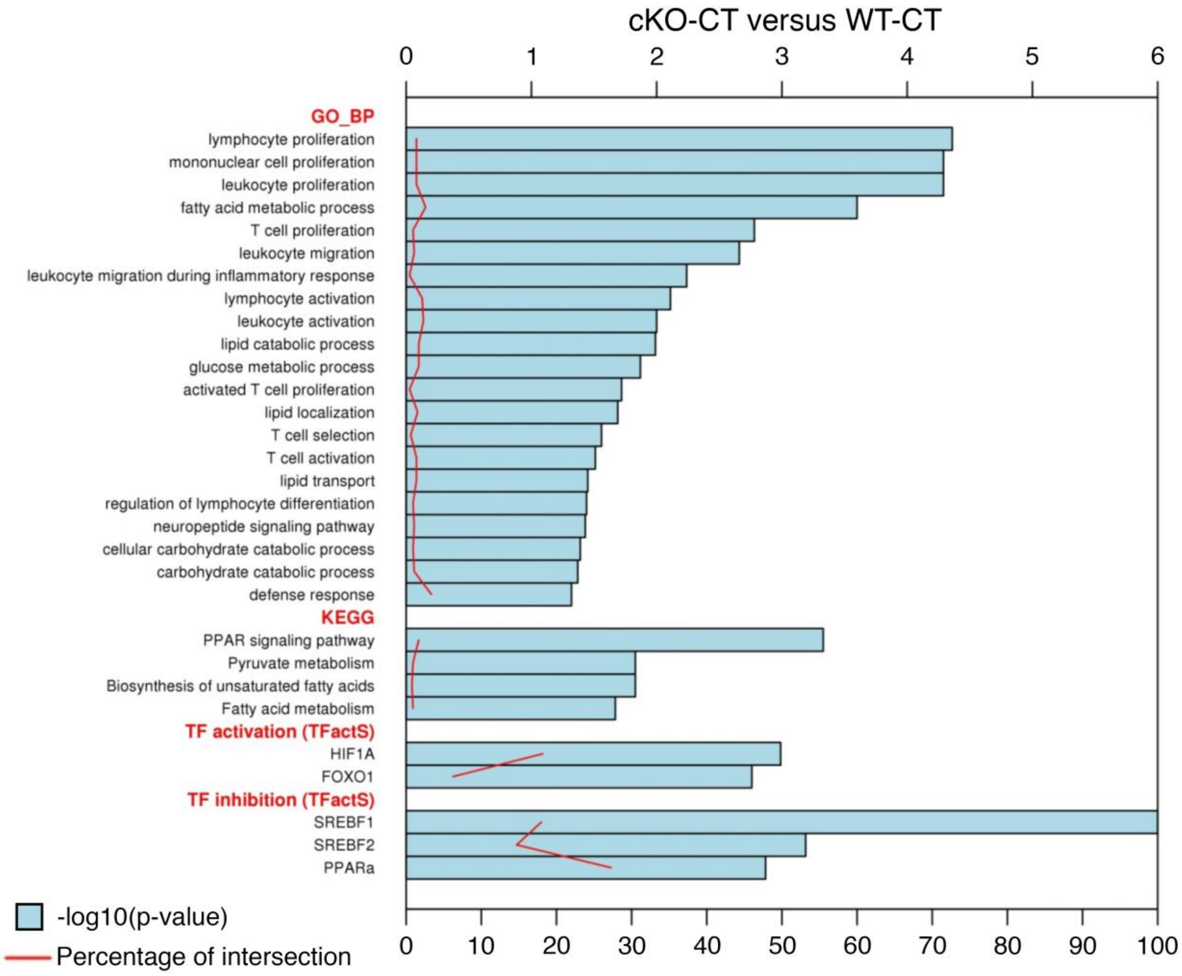

Microarrays analysis submitted to the bioinformatics tools DAVID and TFactS. The list of genes that were significantly modified in cKO-CT mice compared to WT-CT mice was submitted to DAVID and to TFactS. For DAVID analysis, the terms represented are terms significantly modified ($p<0.05$) and selected from the total DAVID analysis based on the list of genes 1,5 fold up- or downregulated in the cKO-CT mice compared to the WT-CT mice. The % of intersection is calculated as the % of genes regulated in cKO-CT mice compared to WT-CT mice on the total target genes of the transcription factor present in the database of TFactS (www.tfacts.org)[1]. The regulation type of transcription factors (activation or inhibition) is indicated if it was found significant ($P < 0.05$) by TFactS (sign-sensitive).

Supplementary Fig. 5

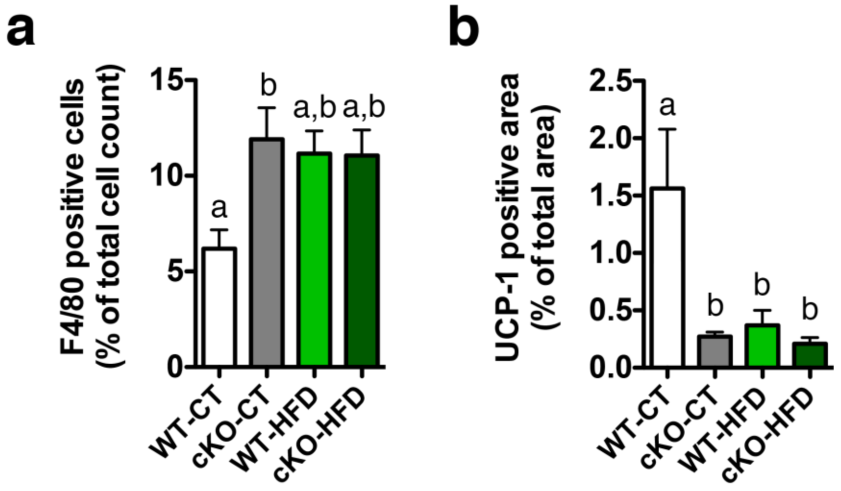

Adipose tissue *Nape pld* deletion induces macrophage infiltration and decreases browning. (**a**) F4/80 positive cell count (n=6-10). (**b**) UCP1 positive cell count (n=6-10). Data are shown as the means ± SEM. Data with different superscript letters are significantly different ($P < 0.05$) according to post hoc ANOVA one-way.

Supplementary Fig. 6

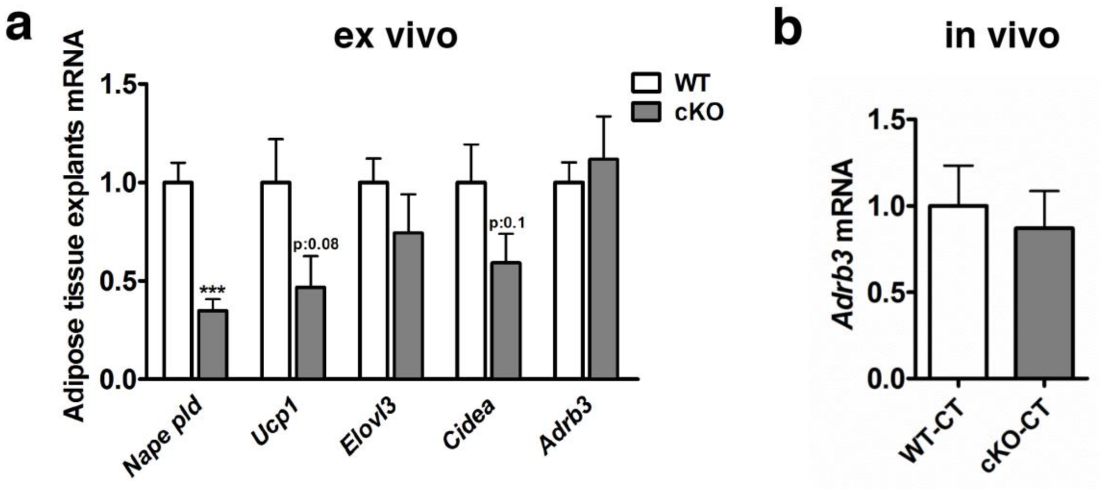

Browning phenotype is mainly conserved in *ex vivo* explants of adipose tissue's *Nape pld* deleted mice. (a) mRNA expression of *Nape pld*, *Ucp1*, *Elovl3* and *Cidea* in 24h cultured adipose tissue explants from WT and cKO mice. (**b**) *Adrb3* mRNA expression in subcutaneous adipose tissue of WT and cKO mice. *** indicates a significant difference (P < 0.01) versus WT-CT according to the unpaired two-tailed Student t-test.

Supplementary Fig. 7

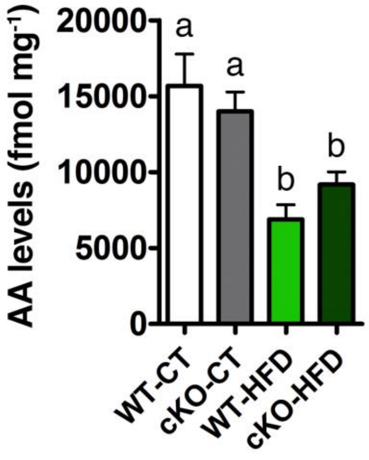

Adipose tissue *Nape pld* deletion impacts on eicosanoids metabolism. SAT arachidonic acid (AA) content measured by LC-MS/MS in fmol/mg tissue (n=10). Data are shown as the means ± SEM. Data with different superscript letters are significantly different ($P < 0.05$) according to post hoc ANOVA one-way.

Supplementary Fig. 8

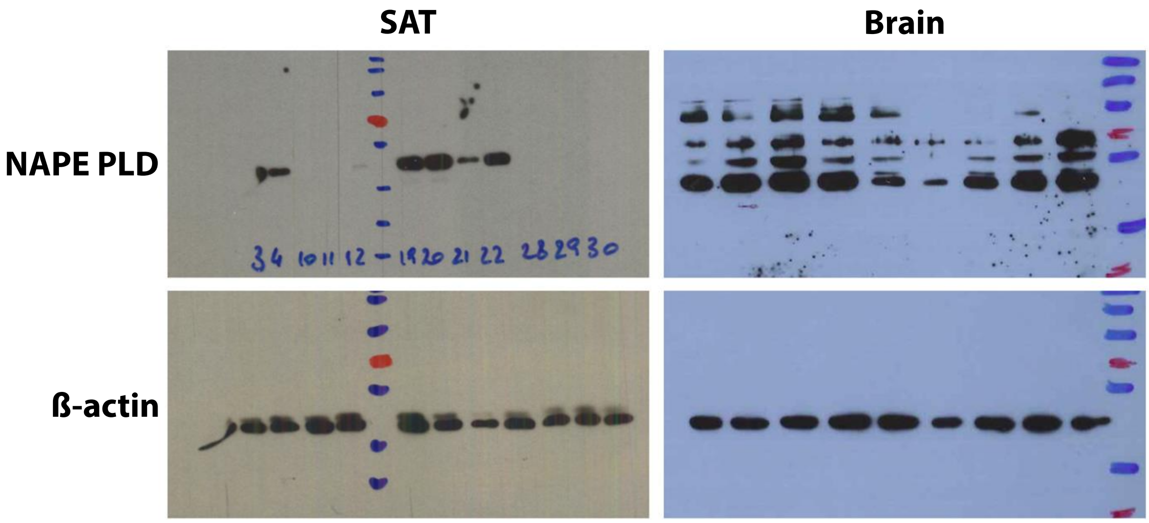

Full and unedited blots for Figure 1 (left) and Supplementary Figure 2 (right). NAPE PLD has size 46 kD, β-Actin has size 43 kD.

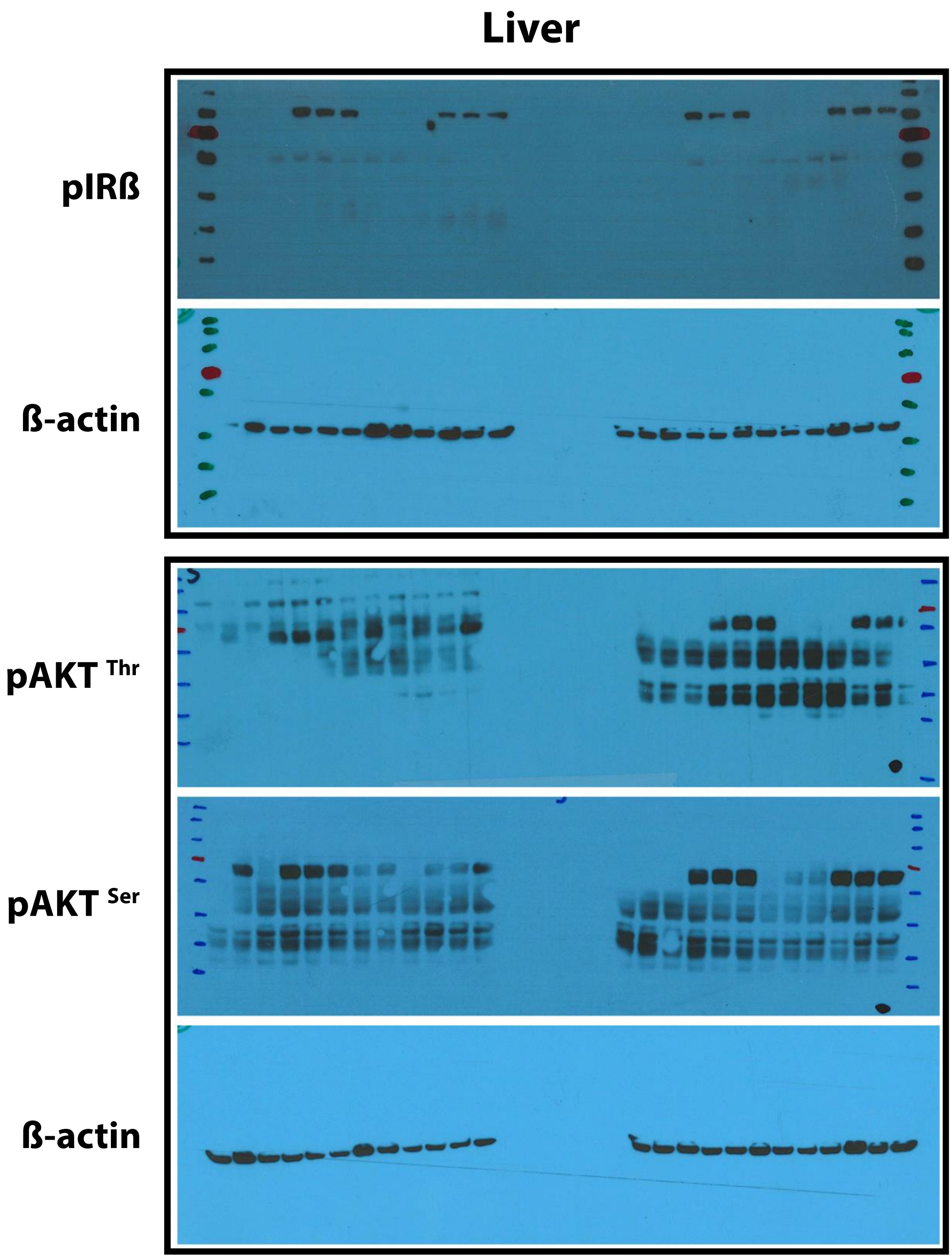

Full and unedited blots for Figure 3 (liver samples). Following molecular weights were predicted: pIRß: 95 kD, ß-actin: 43 kD, pAKT: 60 kD.

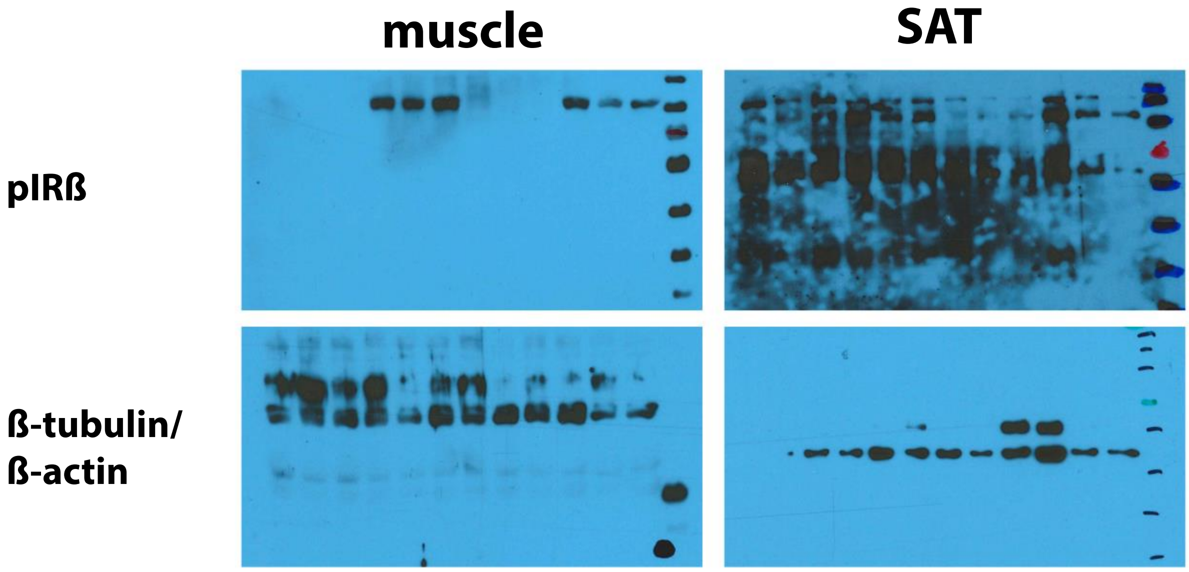

Full and unedited blots for Figure 3 (muscle and subcutaneous adipose tissue samples). Following molecular weights were predicted: pIRß: 95 kD, ß-actin: 43 kD, ß-tubulin: 50 kD.

<u>Supplementary tables</u>

Supplementary Table 1: Phospholipids analysis in SAT.

Phospholipids (pmol/mg)	WT-CT	cKO-CT	WT-HFD	cKO-HFD
**PC aa C36:4**	100.72±17.49	80.1±19.75*	36.36±11.53•	53.94±32.01•
**PC aa C40:4**	1.88±0.48	1.36±0.48*	1.03±0.3*	1.46±0.83
**PC aa C40:7**	4.01±0.42	3.49±0.47*	1.55±0.38•	2.49±2.12*
**PC ae C32 :0**	6.15±1.24	4.59±0.89*	2.43±0.33•	2.99±1.03•
**PC ae C36 :4**	3.23±0.8	2.21±0.69*	1.25±0.4•	1.52±0.65•
**PC ae C38 :5**	3.93±1.19	2.8±0.85*	2.27±0.62•	2.89±1.37*
**PC ae C40 :5**	0.71±0.14	0.55±0.14*	0.38±0.09•	0.46±0.24*
**LPE a C18 :2**	0.59±0.17	0.44±0.14*	0.18±0.05•	0.19±0.07•
**LPE a C20:4**	0.33±0.14	0.23±0.08*	0.1±0.03•	0.12±0.04•
**PE aa C22:2**	0.01±0.002	0.008±0.002*	0.005±0.001•	0.005±0.002•
**PE aa C40:4**	4.66±1.53	5.94±2.17*	4.71±1.09•	5.62±2.55*
**PE ae C42:1**	0.83±0.12	0.7±0.17*	0.42±0.11•	0.48±0.21•
**SM C16:0**	126.04±30.91	98.48±15.03*	82.05±5.46•	90.48±19.13*
**SM C16:1**	5.063±0.75	4.39±0.44*	4.78±0.56	5.00±0.85
**SM C17:0**	1.96±0.29	1.63±0.15*	2.89±0.34•	3.07±0.56•
**SM C18:0**	36.2±7.74	28.85±5.25*	51.78±4.78•	49.75±8.01•
**SM C18:1**	5.82±1.9	4.56±0.87*	10.97±2.92•	9.82±2.15•
**SM C20:2**	0.89±0.32	0.64±0.21*	0.31±0.04•	0.43±0.24•
**SM C22:2**	2.75±1.38	1.24±0.7*	0.91±0.56*	1.59±1.2
**SM C24:4**	1.28±0.44	0.78±0.42*	0.52±0.34*	0.65±0.44*

***PC aa**: Phosphatidylcholines with diacyl bonds; **PC ae**: Phosphatidylcholines with acyl/ether bonds; **LPC a or** e: Lysophosphatidylcholines with acyl or ether bonds; **LPE a or e**: lysophosphatidylethanolamines (acyl or ether bond); **PE aa**: Phosphatidylethanolamines with diacyl bonds; **PE ae**: Phosphatidylethanolamines with acyl/ether bonds; **SM**: sphingomyelines.*

Phospholipids significantly modified in cKO-CT mice compared to WT-CT mice. Values are expressed in pmol/mg tissue and are obtained from ESI-MS/MS analysis (n=10). Data are shown as the means ± SEM. * indicates a significant difference ($P < 0.05$) versus WT-CT, • indicates a significant difference ($P < 0.05$) versus WT-CT and cKO-CT according to post hoc ANOVA one-way.

Supplementary Table 2: Primers sequences

Primers	Forward Sequence	Reverse Sequence
Acaca	GTTGAGACGCTGGTTTGTAGAA	GTCCTTATTATTGTCCCAGACGTA
Acox	CTATGGGATCAGCCAGAAAG	AGTCAAAGGCATCCACCAAAG
Acsl1	TGGGGTGGAAATCATCAGCC	CAC GC TT CA AC GTACA
Adrb3	CGACATGTTCCTCCACAAATCA	TGGATTCCTGCTCTCAAACTAACC
Cd11c	ACGTCAGTACAAGGAGATGTTGGA	ATCCTATTGCAGAATGCTTCTTTACC
Cd206	CCTCTGGTGAACGGAATGAT	CTTCCTTTGGTCAGCTTTGG
Cd3g	CCATCTCAAAGGAAACCAAC	TCTCTACTGGGCTCTCTCCAA
Cd68	CTTCCCACAGGCAGCACAG	AATGATGAGAGGCAGCAAGAGG
Cidea	GCAGCCTGCAGGAACTTATC	TCATGAAATGCGTGTTGTCC
Elovl3	TTCTCACGCGGGTTAAAAATG	GACCAACAGATAGACGACCAC
F4/80	TGACAACCAGACGGCTTGTG	GCAGGCGAGGAAAAGATAGTGT
Fasn	TTCCAAGACGAAAATGATGC	AATTGTGGGATCAGGAGAGC
Il1b	TCGCTCAGGGTCACAAGAAA	CATCAGAGGCAAGGAGGAAAAC
Lbp	GTCCTGGGAATCTGTCCTTG	CCGGTAACCTTGCTGTTGTT
MCP1	GCAGTTAACGCCCCACTCA	CCCAGCCTACTCATTGGGATCA
Napepld	TTCTTTGCTGGGGATACTGG	GCAAGGTCAAAAGGACCAAA
PAI1	ACAGCCTTTGTCATCTCAGCC	CCGACACAAAGAAGGA
PPARa	CAACGGCGTCGAAGACAAA	TGACGGTCTCCACGGACAT
Ppargc1a	AGCCGTGACCACTGACAACGAG	GCTGCATGGTTCTGAGTGCTAAG
Prdm16	TCAGGAAGGGGAAGGAGAGA	ACGGCTTCTCTTTGTTGTGC
RPL19	GAAGGTCAAAGGGAATGTGTTCA	CCTTGTCTGCCTTCAGCTTGT
Ucp1	GCT ACA CGG GGA CCT ACA ATG	CGTCATCTGCCAGTATTTTGTT

Supplementary Table 3: Phyla enriched or depleted in cKO-CT mice compared to WT-CT mice.

Phyla	WT-CT		cKO-CT		cKO-CT vs WT-CT	
	**Mean**	**SEM**	**Mean**	**SEM**	**p-value**	**Changes (%)**
**Actinobacteria**	0,56	0,23	0,04	0,01	0,01	**-91,98**
**Proteobacteria**	7,71	0,45	5,88	0,36	0,007	**-23,69**
**Unclassified**	3	0,33	5,28	0,56	0,01	**76,11**

Data are shown as the means and SEM. Only significant differences ($P < 0.05$) between the two groups are indicated. P values are based on the 2-sample *t*-test assuming equal variances. Student *t*-test results were corrected by an FDR test according to the Benjamini-Hochberg procedure, with an α of < 0.05. No significant p-values after FDR correction.

Supplementary Table 4: Families enriched or depleted in cKO-CT mice compared to WT-CT mice.

Families	WT-CT		cKO-CT		cKO-CT vs WT-CT	
	**Mean**	**SEM**	**Mean**	**SEM**	**p-value**	**Changes (%)**
Alcaligenaceae	2,88	0,93	0,52	0,14	0,006	**-81,94**
Bacteroidaceae	2,86	0,4	1,17	0,12	0,0002*	**-59,02**
Clostridiaceae	0,01	0,01	0,09	0,03	0,04	**933,89**
Coriobacteriaceae	0,56	0,23	0,04	0,02	0,01	**-92,15**
Erysipelotrichaceae	9,35	3,04	2,8	0,41	0,01	**-70,06**
Lactobacillaceae	0,12	0,04	0,02	0,01	0,006	**-85,09**
unclassified_Bacteria	3	0,33	5,28	0,56	0,01	**76,11**
unclassified_Clostridia	0,34	0,06	0,84	0,16	0,04	**144,41**
unclassified_Clostridiales	7,17	0,77	9,19	0,57	0,05	**28,15**

Data are shown as the means and SEM. Only significant differences ($P < 0.05$) between the two groups are indicated. P values are based on the 2-sample *t*-test assuming equal variances.
* Student *t*-test results were corrected by an FDR test according to the Benjamini-Hochberg procedure, with an α of < 0.05. *: significant p-values after FDR correction.

Supplementary Table 5: Genera enriched or depleted in cKO-CT mice compared to WT-CT mice.

Genera	WT-CT		cKO-CT		cKO-CT vs WT-CT	
	**Mean**	**SEM**	**Mean**	**SEM**	**p-value**	**Changes (%)**
Allobaculum	7,61	2,47	2,3	0,34	0,01	**-69,72**
Bacteroides	2,86	0,4	1,17	0,12	0,0002*	**-59,02**
Clostridium	0,01	0,01	0,09	0,03	0,04	**925,01**
Lactobacillus	0,12	0,04	0,02	0,01	0,006	**-85,09**
Odoribacter	2,63	0,45	4,48	0,37	0,008	**70,65**
Olsenella	0,45	0,21	0	0	0,01	**-99,65**
Parabacteroides	0,67	0,15	0,33	0,07	0,03	**-50,78**
Parasutterella	2,88	0,93	0,52	0,14	0,006	**-81,94**
unclassified	6,88	0,86	4,51	0,22	0,005	**-34,51**

Data are shown as the means and SEM. Only significant differences ($P < 0.05$) between the two groups are indicated. P values are based on the 2-sample *t*-test assuming equal variances.
* Student *t*-test results were corrected by an FDR test according to the Benjamini-Hochberg procedure, with an α of < 0.05. *: significant p-values after FDR correction.
